# Telemonitoring system for patients with chronic kidney disease undergoing peritoneal dialysis: Usability assessment based on a case study

**DOI:** 10.1371/journal.pone.0206600

**Published:** 2018-11-06

**Authors:** Marcos Antonio Martínez García, Martha Socorrro Fernández Rosales, Eduardo López Domínguez, Yesenia Hernández Velázquez, Saúl Domínguez Isidro

**Affiliations:** 1 General Hospital of Zone No. 11,Instituto Méxicano del Seguro Social, Xalapa, Veracruz, Mexico; 2 Department of Computer Science, Laboratorio Nacional de Informática Avanzada, Xalapa, Veracruz, México; University of Wisconsin, UNITED STATES

## Abstract

There are two million people with chronic kidney disease (CKD) worldwide. In Mexico, it is estimated that by 2025, there will be 212 thousand CKD cases. Among the renal replacement treatments, peritoneal dialysis (PD) exists either in the continuous ambulatory (CAPD) or automated (APD) mode, which requires continuous monitoring and strict control. Thus, several software systems have been proposed to perform reliable remote monitoring of patients using PD but also to achieve the goal with effectiveness, efficiency and satisfaction; i.e., in software engineering, this is called usability. However, few studies have addressed usability issues using case studies with patients and medical staff in real domains. In this paper, we present a usability assessment of a telemonitoring system for patients with CKD on peritoneal dialysis treatment through a case study with patients and medical staff of the Mexican Institute of Social Security (IMSS). The usability evaluation was carried out through the application of two satisfaction instruments. These instruments evaluated multiple usability criteria, such as navigability, interactivity, motivation, satisfaction, and applicability. The results obtained from the usability evaluation show that, on average, the services offered by the system have 91.3% acceptance by users (patient-doctors), with the APD and CAPD exchange data registration services having the highest acceptance for patients, with a positive perception of 94.5% and 92.3%, respectively. Meanwhile, for the doctors and nurses, the alarm reception for patients in a risk situation was highest with 95% acceptance. Based on the obtained results, the evaluated telemonitoring system holds wide acceptance, satisfaction, and applicability from patients’ and doctors’ perspectives. It is also noted that the evaluated system considers and satisfies the requirements and suitable parameters that should be monitored in PD treatment according to studies presented in the literature.

## Introduction

Worldwide, there are two million people with chronic kidney disease (CKD) [1]. In Mexico, the problem of terminal CKD presents alarming dimensions since based on projections, it is estimated that by 2025, there will be 212 thousand cases [1]. This disease in the Mexican context is classified as an emerging disease because of the growing number of cases, including approximately 80,000 patients in the IMSS who receive, among the renal replacement therapy PD options, continuous ambulatory (CAPD) and automated (APD) [2–4] PD. Both PD modalities must be accompanied by a diet to control the levels of phosphorus, potassium, sodium, and calcium, as well as restrictions in fluid intake [5]. In these renal replacement treatments, approximately 70% of patients attend the IMSS [2]. CAPD and APD are home therapies, carried out throughout the day, and it is only necessary to go to the hospital to carry out tests and reviews, which usually occur every three or four months [6]. The main PD disadvantages consist of the fact that the patient or a caregiver should perform the treatment. In addition, it requires careful dedication when taking biomedical data and carrying out analytical work to determine dehydration or fluid retention in the body [5]. The patient also evaluates and records several types of data on a dialysis sheet, including the dialysis total time, infused PD fluid volume, infusion time per cycle, dwell time, drained fluid characteristics (clear, cloudy, other aspects), and assessment of pain presence and/or discomfort. These data, recorded on paper, are delivered for review every three months. During this period, there is no biomedical index monitoring or rapid risk situation control in cases of an imbalance or delay in the limit range provided by the physician. Additionally, illegibility problems in the patient's notes should be considered, as well as the notes being lost or forgotten. Some studies [7–12] have proposed systems to address this problem. However, the telemonitoring system presented by Cuevas JR et al. [13] is characterized by offering different services for monitoring, control and remote treatment of patients on CAPD and APD. This system is assembled by a native Android application, which provides services to the patient, and by a mobile Web application, which provides a set of services to the medical staff. In this context, usability is one of the central requirements in software quality terms that this type of system must provide [14]. The ISO 9241–11 standard defines usability as the capability of a product to be used by certain users to achieve specific objectives with effectiveness, efficiency, and satisfaction within a specific use context [14]. The evaluation of the systems' usability is a tool that allows the users to identify the most important characteristics that are qualified when using the applications and, in turn, provides information about aspects that should be improved in the system to increase the possibility of its use in the daily lives of people. There are different studies in the literature [15, 16] that identify the lack of research focused on evaluating the usability of telemonitoring systems through case study series with patients on PD treatment and physicians in a real environment [15–20].

This article presents a usability evaluation conducted on a telemonitoring system, proposed by Cuevas et al. [13], for patients with chronic kidney disease in peritoneal dialysis treatment through a case study with patients and medical staff of the IMSS. To achieve this, the usability evaluation was carried out on the main services offered in the mobile applications for patients and physicians belonging to the telemonitoring system proposed in [13]. In our study, we perform a usability evaluation method composed of two questionnaires or satisfaction instruments aimed at the end users of the system. This evaluation was due to the possibility of having patients and medical staff committed to interacting and using the system as a tool that complements the follow-up and control of PD treatment. The results obtained show that the APD and CAPD exchange data registration services had the highest acceptance in terms of usability among patients with 94.5%. On the other hand, the results obtained in the usability evaluation of the services offered to the physician reflect that the highest percentage of positive qualifications (95%) was obtained in the alarm reception service for patient risk situations. Finally, it is noted that the telemonitoring system [13] evaluated in this study considers and satisfies the ideal requirements and parameters that should be monitored in the PD treatment according to the study presented by Nayak et al. [21].

## Materials and methods

### Case study description

Within the format of a case study, a descriptive, prospective, longitudinal and observational analysis was conducted regarding a telemonitoring system for patients with CKD under dialysis support, as proposed by Cuevas et al. [13]. To achieve this goal, systems engineers from the National Laboratory on Advanced Informatics (LANIA) were trained in the management, monitoring, and registration of dialytic therapy of patients on PD at the General Hospital of Zone No. 11 of Xalapa, Veracruz, Mexico. The evaluated system allows, through a mobile web application and a native application in Android 4.0 or higher, patients to interact with doctors and nurses remotely regarding their symptoms, signs, and conditions of their dialysis therapy, as well as identifying risk situations through alert generation, which may avoid hospital admissions to emergency units. To prove the system usability, tests were carried out under fictitious cases, and then the case study was carried out by means of the selection for convenience of a sample obtained from the population of patients assisted by the PD program (CAPD and APD), with the approval of the National Research Commission Scientific (Ethical Committee) of the IMSS and the signing of informed consent by participants. Selection criteria included the availability of the patient and the relatives to receive training in the system management, facility of use of mobile devices with Android 4.0 or higher and an Internet connection. Twenty-four patients, 15 on CAPD and 9 on APD, participated in the case study together with four nephrologists, one internist and two specialist nurses. After training the physicians on the mobile web application, the patients were telemonitored using the system for a period of 9 months in order to reach an average monitoring time of five months/patient. Twenty-four subjects completed the monitoring, applying two usability evaluation instruments to patients, patients' relatives, doctors, and nurses.

### Development of usability evaluation instruments

According to the Nielsen decalogue [22], the best practice guide of mobile web application development [23] and the related works analysis [15–20], diverse criteria for the usability evaluation of the telemonitoring system were identified and are detailed below (see Table 1).

**Table 1:** Selected criteria based on specialized literature for the usability evaluation of the telemonitoring system.

Usability criteria	Description
System visibility	It is the property of keeping the user permanently informed about what is happening when interacting with a system.
Congruence between the real world and the system	The system should interact with the user in the user's language, considering both the text and the execution order of actions.
User control and freedom	The user should be able to navigate freely, easily find outlets and alternative routes and have all the facilities he needs to operate the system.
Consistency and standards	A system must follow a uniform standard in terms of functioning and terminology in all its elements.
Prevention of errors by design	It is the property of validating all the system processes to avoid errors during user interactions
Recognition instead of memory	The user should have all the information available to perform tasks without using his memory during the interaction sequence.
Flexibility and efficiency of use	A flexible user interface provides the necessary resources to perform tasks regarding the type of user and disposition.
Minimalistic and esthetic design	Each remaining element in the user interface should focus attention on text or images (visual design) that are important for operations.
Handling errors efficiently	Error messages should be written in a language that the user can understand, without technicalities, and should suggest a solution or a way out.
Help documentation	It is suggested that this information is easy to find and is oriented to tasks performed by the user.
Aim and purpose clarity	The user must know the objectives of each interface and service offered by the system.
Navigability	It includes clear and consistent navigation options and unambiguous links.
Interactivity	It is necessary that the user interfaces are interactive so that they facilitates field and service understanding.
Activities	They should be in accordance with the service provided, as well as sufficient and complete for the service purposes. Furthermore, the activities must be clear and significant for each domain.
Feedback	It is supported by accurate information, correct answers, contents or important concepts.
Motivation	The system must have an orientation to intrinsic and extrinsic goals. The patient should feel stimulated using the system.
Satisfaction	The set of characteristics that make the system easy to use and pleasant for the user, and for that reason, the user prefers and recommends it among other similar systems.
Applicability	Refers to user perceptions about the system usage or application in their daily life.

Considering the described criteria, in our study, two instruments were constructed for evaluating the usability of the telemonitoring system introduced in [13]:

1. The instrument for evaluating the patient's native application services is included later in this document as Instrument 1.
2. The instrument for evaluating the medical personnel's mobile web application services is included later in this document as Instrument 2.

Moreover, these instruments allowed us to obtain feedback regarding the design and functionality of the telemonitoring system.

#### Description of instrument 1

Instrument 1 was designed to evaluate the usability of the following services of the patient's native Android application [13]:

1. APD and CAPD exchange data registration services. These services allows the patient to enter the necessary biomedical data that manage the monitoring and control of dialysis under the CADP and ADP modalities.
2. Alerts. This service offered to the patient informs the doctor, by a text message and an email, concerning a patient's possible risk situation, such as the characteristic of drained fluid being different from transparent or ultrafiltration not being as expected.
3. Notifications search. This service allows patients to examine the dates of events related to their PD treatment, such as medications, reminders and doctor's recommendations.

According to the services mentioned above, Instrument 1 is composed of three sections. Section 1 includes 39 questions that evaluate the usability of the APD and CAPD exchange data registration services. Section 2, a set of 14 questions, evaluates the usability of the alert service. Finally, section 3, composed of 35 questions, evaluates the notifications search service.

#### Description of instrument 2

Instrument 2 was designed to evaluate the usability of the following services of the doctor's mobile Web application [13]:

1. APD and CAPD exchange data registration search services. In these services, the medical staff can monitor different data of the patient's treatment, such as ultrafiltration, partial balance, total balance, type of fluid and abnormal symptoms (dizziness, vomiting, swelling, etc.) that the patient reports.
2. Alerts search. In this option, the physician can consult the alert details generated by the patients
3. Notifications. By this service, the doctor can generate a message to the patient regarding the following situations: a recommendation in response to a received alert, a reminder of an appointment, or a recommendation of adjusting the patient’s therapy and/or medication.

Therefore, Instrument 2 is composed of three sections. Section 1 includes 36 questions that evaluate the usability of the APD and CAPD exchange data registration search services. On the other hand, the 2nd and 3rd sections evaluate the usability of the alerts search service and notifications, respectively. Both sections are composed of 34 questions.

## Results

### Sociodemographic and clinical results

In this case, we analyzed sociodemographic and clinical information of the patients' sample, 12 men and 12 women with a mean age of 53.4 years. In addition, 46% of them have a basic educational level, 25% have a medium educational level, and 29% have a high educational level (see Table 2). These patients had DP treatments for 43.3 months on average before starting the case study, where 71.4% had an assisted process and 28.6% were self-sufficient. During the telemonitoring process and control of the patients on PD treatment through the system by health staff, several risk situations were immediately detected, such as fluid retention, dehydration, and peritonitis. Moreover, it is highlighted that the average effective use time of the application by patients was 4.9 months/patient (see Table 3).

**Table 2:** Sociodemographic characteristics of the patients' sample (n = 24).

Gender		Age/years		Education Level	
Women	12 (50%)	53.4 ± 14.24 (CI 59.43–63.52)		Basic	11 (45.83%)
Men	12 (50%)	Minimum	20	Medium	6 (25.00%)
		Maximum	67	High	7 (29.16%)

where CI: Confidence interval

**Table 3:** Clinical characteristics of the patient sample (n = 24).

Characteristic	Criteria	Values
Months in dialytic treatment upon admission	43.29 ±23.19 (CI 33.49–53.08)	
	Minimum	16
	Maximum	90
Causes of terminal renal disease	DM	16 (66.66%)
	Others	4 (16.66%)
	ND	4 (16.66%)
Dialytic process	Assisted	20 (71.42%)
	Self-sufficient	4 (28.57%)
Peritonitis	Yes	3 (12.10%)
	No	21 (87.50%)
Dialytic treatment mode	CAPD	15 (62.5%)
	APD	9 (37.5%)
Effective use time of monitoring in months/patient	4.90±2.66 (CI 3.79–6.05)	
	Minimum	1.2
	Maximum	8.7

where DM: Diabetes mellitus; Other: hypertension, obstructive uropathy, nephroangiosclerosis, polycystic kidneys; ND: Not determined; Assisted: Performed by a relative or caregiver assisting PD patients; CAPD: Continuous ambulatory peritoneal dialysis; APD: Automated peritoneal dialysis; CI: Confidence interval.

### Instrument results

In the developed instruments, the assessment scale selected for each criterion consists of four conceptual levels of satisfaction: a) Strongly agree, b) Moderately agree, c) Somewhat agree and d) Neutral, based on the Likert scale [24]. To simplify the results' presentation and provide a general idea of the bias that the perception of the patients and medical staff involved in the case study takes, a dichotomy is considered on the assessment scale. Therefore, the responses Strongly Agree and Moderately Agree were grouped as positive or favorable rating responses, whereas Somewhat Agree and Neutral were grouped as negative or unfavorable rating responses. The instrument results are presented below.

#### Instrument 1 results

**Fig 1:**
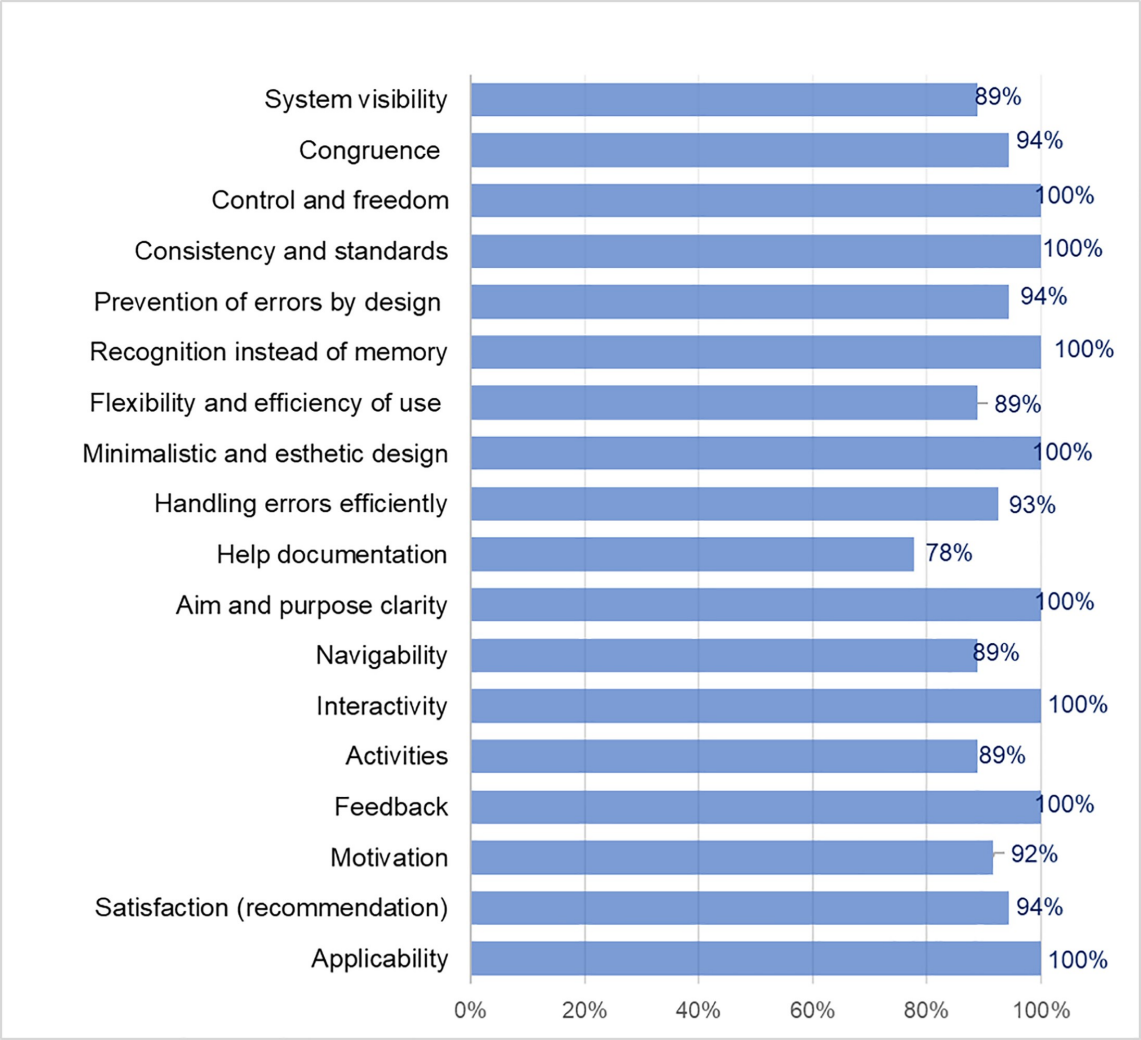
Usability criteria results of the APD and CAPD exchange data registration services for patients on PD treatment.

In general, the CAPD and APD exchange data registration services were the most accepted by patients with 94.5%, as shown in Fig 1.

The criteria that obtained 100% favorable opinion were (see Fig 1) control and freedom, consistency and standards, recognition instead of memory, minimalist and aesthetic design, aim and purpose clarity, interactivity, feedback and applicability. In a range of 91% to 94%, favorable evaluations are the criteria of congruence between the real world and the system, prevention of errors by an adequate design, efficiency in error handling, motivation and satisfaction (recommendation). Finally, the system visibility criteria, flexibility and efficiency of use, help documentation, navigability, and activities obtained a favorable evaluation between 78% and 89%.

**Fig 2:**
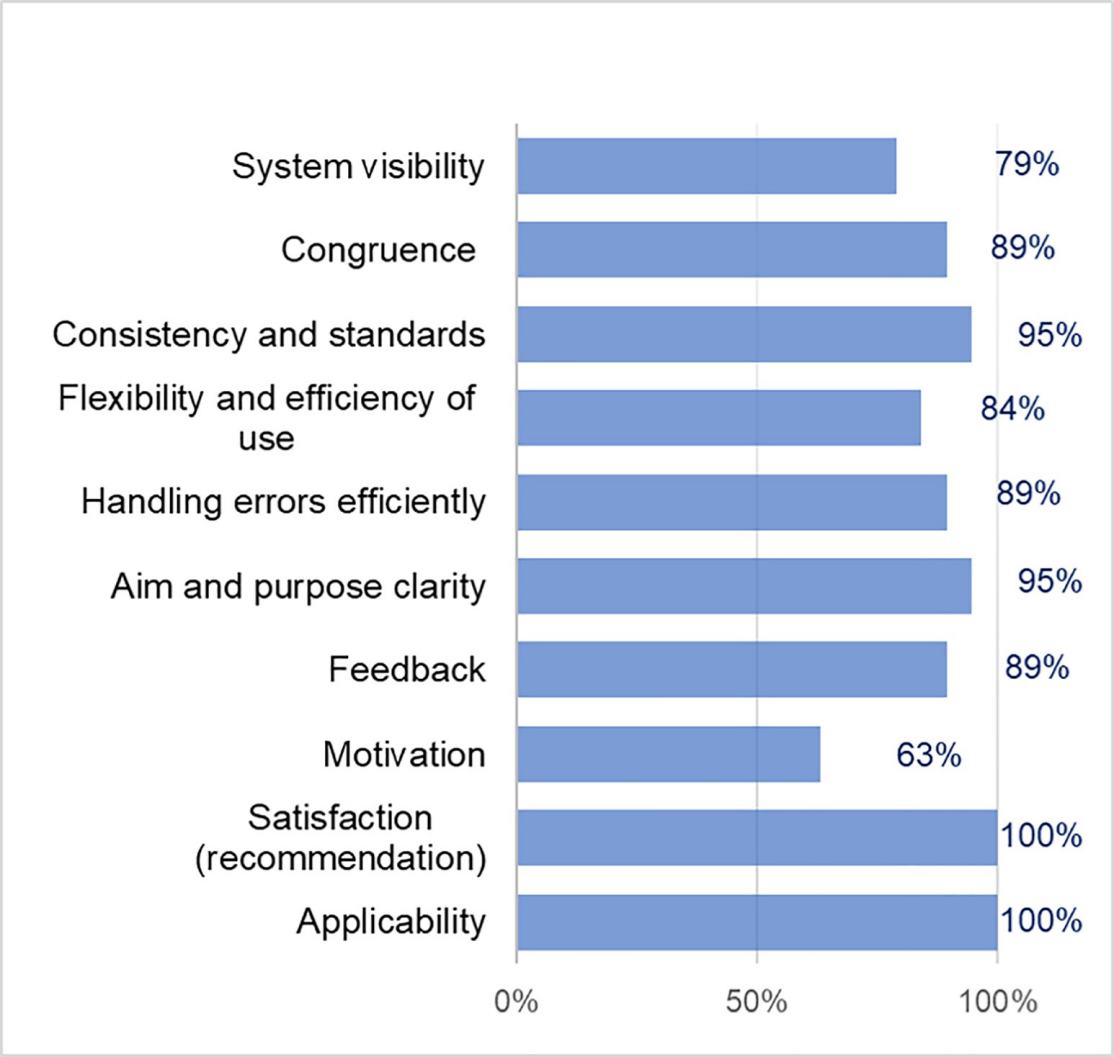
Usability criteria results of the alert generation service.

Regarding the alert generation service, a general percentage of 88% favorable opinions was obtained from the patients. The criteria evaluated with a 100% favorable opinion were satisfaction and applicability (see Fig 2). Moreover, the system visibility criteria, congruence between the real world and the system, consistency and standards, flexibility and efficiency of use, efficiency in handling errors, aim and purpose clarity, and feedback obtained a positive evaluation between 79% and 95%. In this service, the motivation criterion was the one that obtained the lowest percentage of favorable opinion (63%). This was because some patients did not receive feedback from the doctor in response to the alert generated.

**Fig 3:**
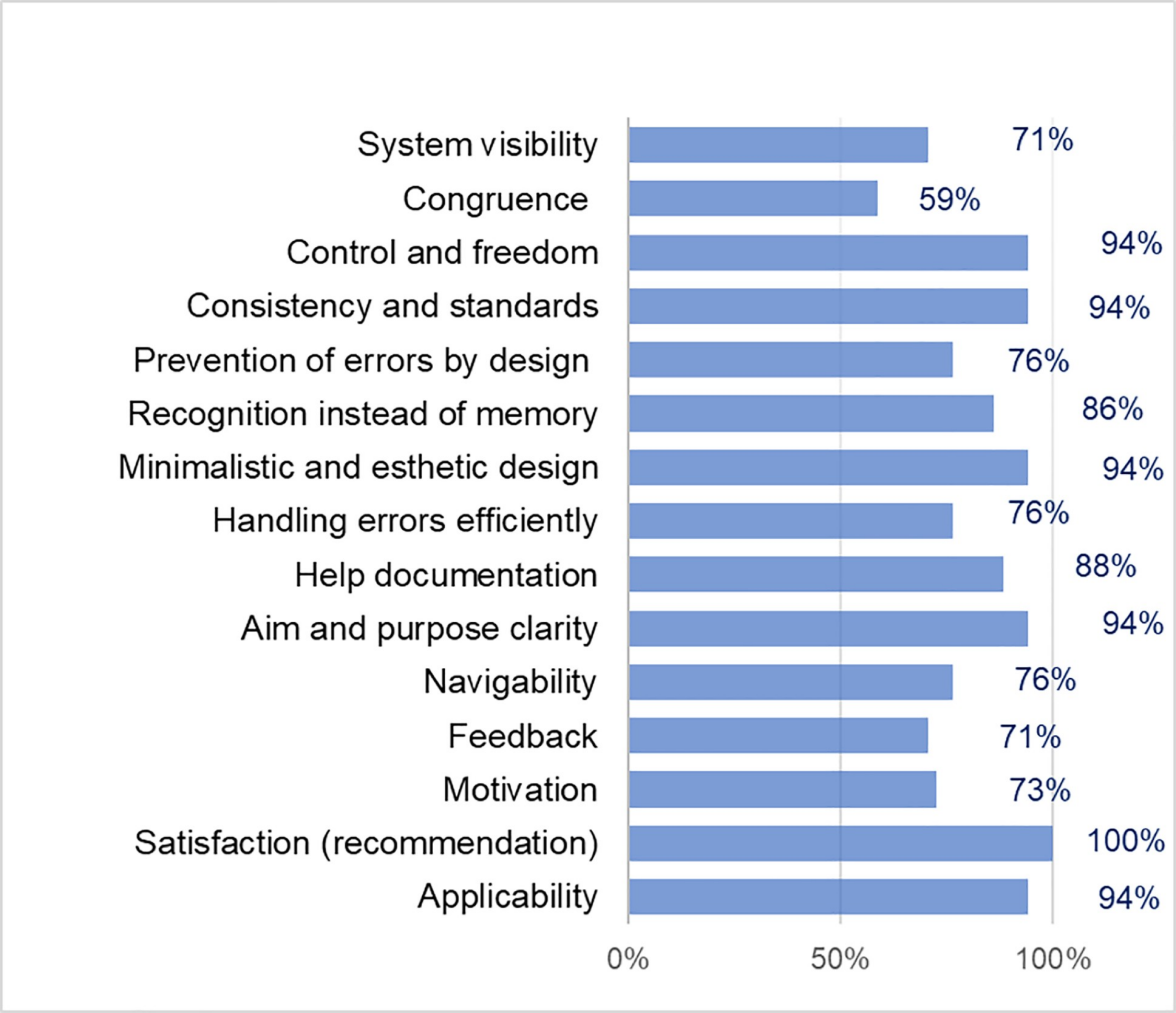
Usability criteria results of the notifications search service.

The notifications search service obtained 83% in the general percentage of positive opinion (see Fig 3). In this service, the satisfaction criterion (recommendation) was the best evaluated with a 100% favorable opinion, and the system visibility criteria, control and freedom, consistency and standards, prevention of errors by an adequate design, recognition instead of memory, minimalist and aesthetic design, efficiency in error handling, help documentation, aim and purpose clarity, navigability, feedback, motivation, and applicability obtained a positive opinion percentage between 71% and 94%. The criterion of congruence between the real world and the system was the lowest in this service, with 59% favorable opinion.

#### Instrument 2 results

**Fig 4:**
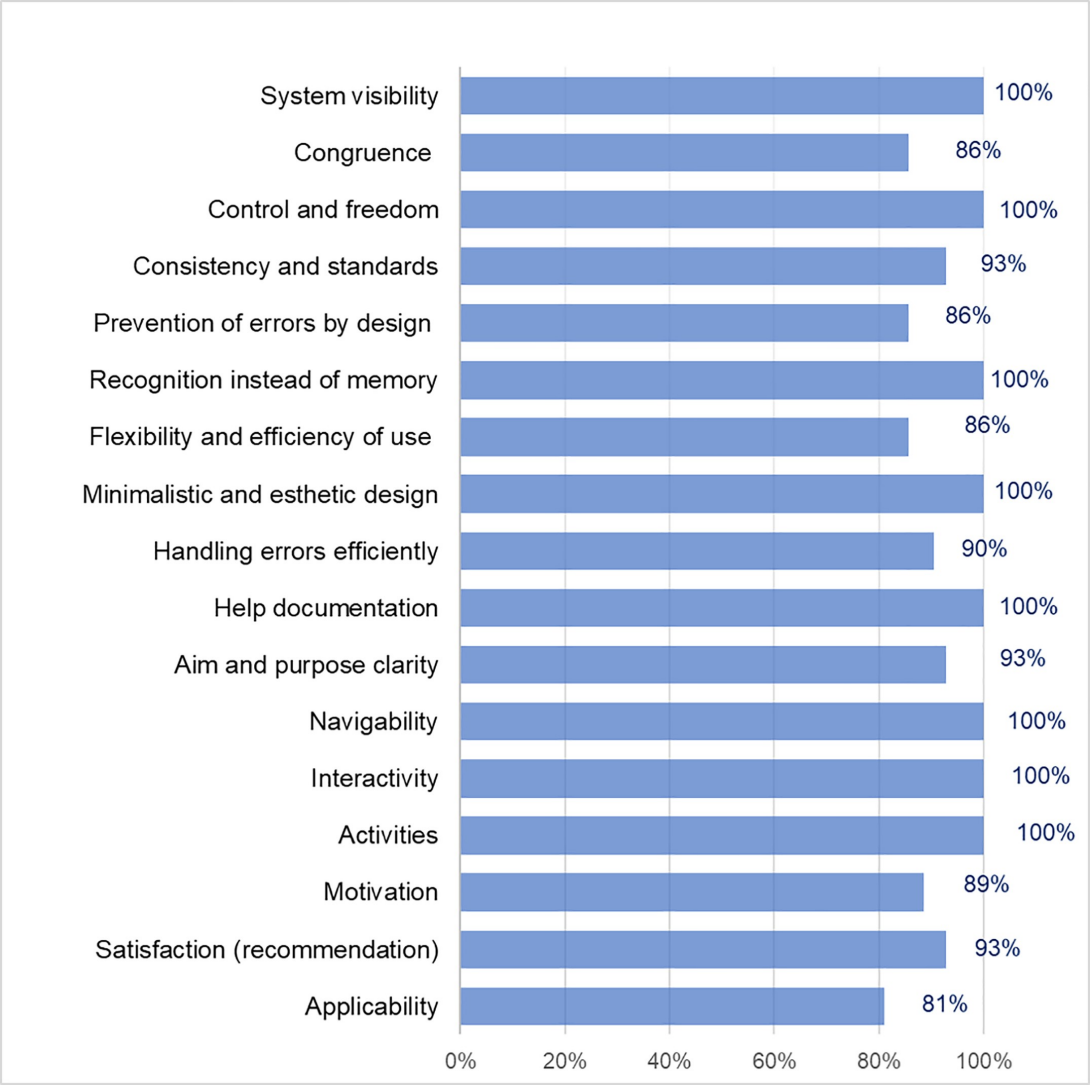
Usability criteria results of the APD and CAPD exchange data registration search services in the mobile web application for medical staff.

The results obtained from Instrument 2, through the medical staff answers in terms of favorable opinion, were 94% for the CAPD and APD exchange registrations search service, 95% for the alerts search service, and 93% for the notification generation service. Concerning the CAPD and APD exchange registration search service, the evaluation criteria with 100% positive opinion were system visibility, control and freedom, recognition instead of memory, minimalist and aesthetic design, help documentation, navigability, interactivity, and activities (see Fig 4). The rest of the evaluation criteria also obtained a satisfactory percentage of favorable opinion between 81% and 93%.

**Fig 5:**
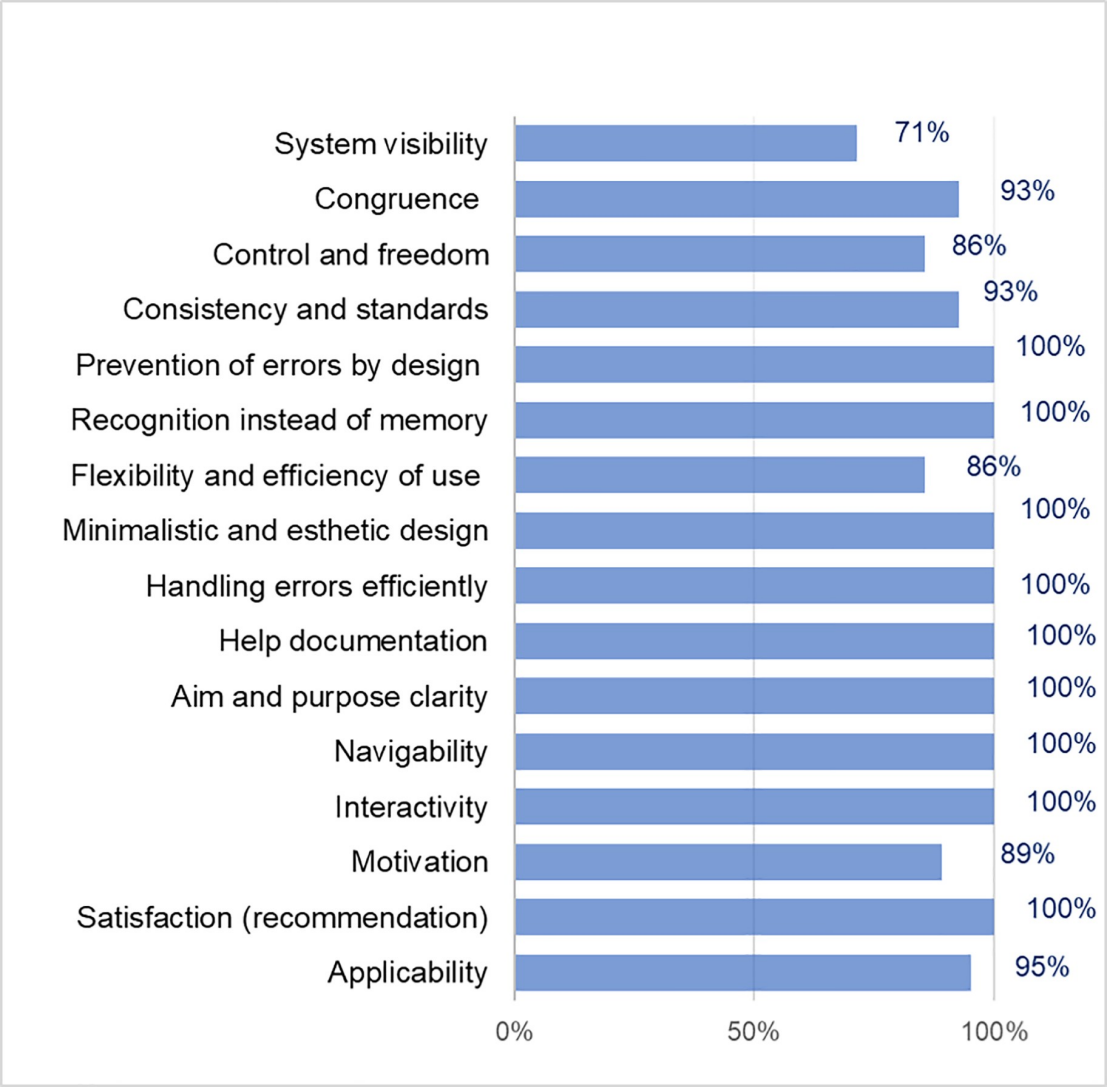
Usability criteria results of the alerts search service in the mobile web application for medical staff.

Regarding the alerts search service, the evaluation criteria with 100% favorable opinion were the prevention of errors by an appropriate design, recognition instead of memory, minimalist and aesthetic design, handling error efficiently, help documentation, aim and purpose clarity, navigability, interactivity, and satisfaction (recommendation) (see Fig 5). The other evaluation criteria are between 71% and 95% favorable opinion.

**Fig 6:**
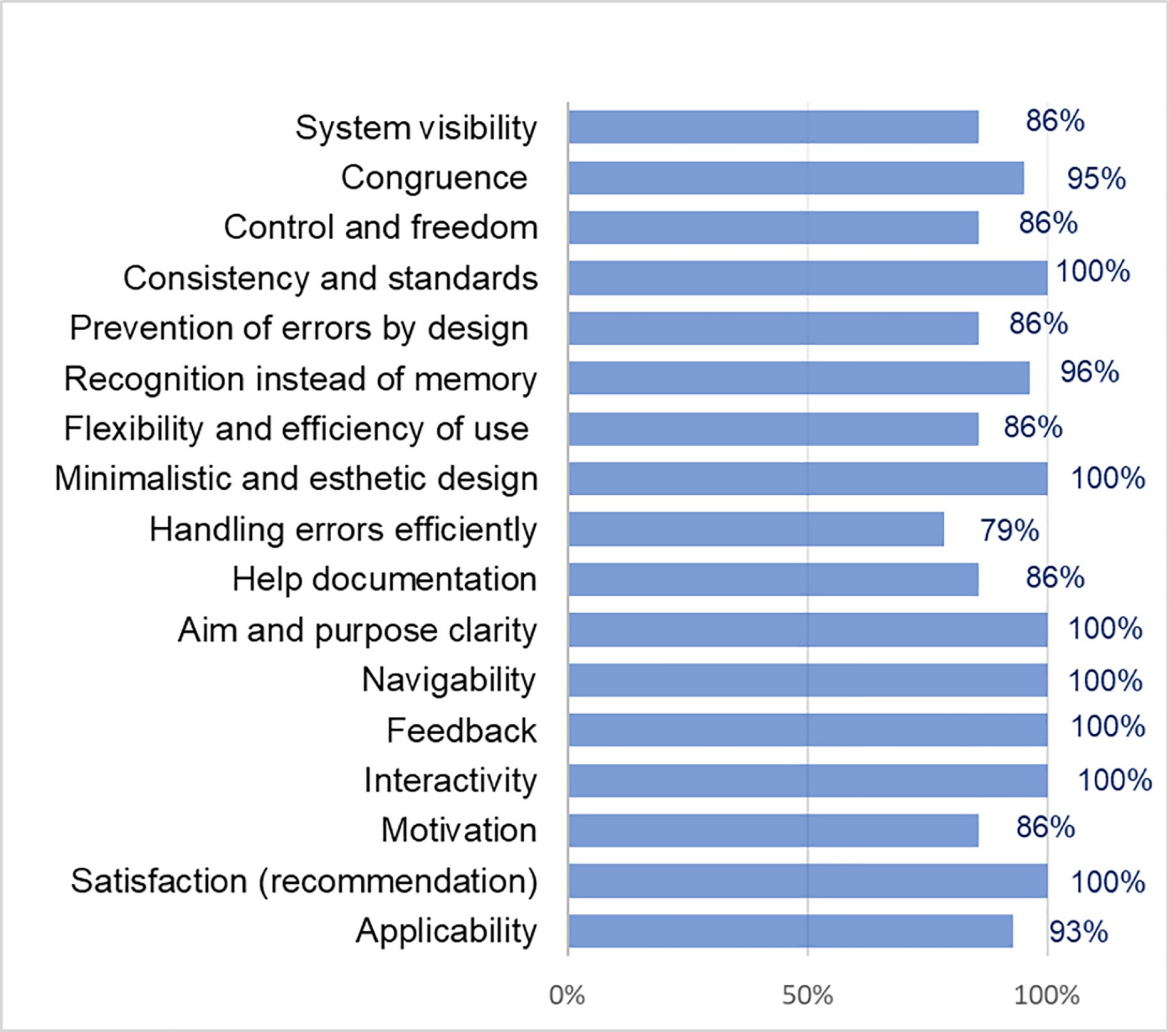
Usability criteria results of the notification generation service in the mobile web application for medical staff.

Finally, within the notification generation service, the evaluation criteria with 100% favorable opinion were consistency and standards, minimalist and esthetic design, aim and purpose clarity, navigability, interactivity, feedback, and satisfaction (recommendation) (see Fig 6). The other evaluation criteria obtained between 79% and 96% favorable opinion.

Overall, it was found that from the patients and doctors' viewpoint: 1) The text, the images, the order and the way in which the information is presented are adequate, allowing a natural relationship between the users (patient and doctor) and the application; 2) both mobile applications of the system follow a consistent standard in all its parts, namely, its design, operation, and terminology; 3) both the patient and the doctor have all the information available and are not forced to use their memory to follow the sequence of the interaction within the services used; 4) the interface is flexible, having the necessary resources to behave according to the needs of both users; 5) there are no distracting text or image elements that are important for operational purposes; 6) the objective pursued by each service is clear to the patient and doctors; 7) within the services, there are clear and consistent navigation options; 8) the patient and doctor feel motivated to use the services; 9) the patient and doctor perceive the services to be easy and pleasant to use; and therefore recommend them to other patients and doctors; and 10) patients and doctors perceive a high utility of the telemonitoring system.

## Discussion

Some studies [15–20] have carried out a usability assessment of systems focused on remote monitoring and control of patients with CKD on PD treatment. In [17], a possible study protocol was introduced, which evaluates the utility and scalability of a telemedicine system for patients with CKD. However, the study did not include results considering a case study in a real scenario. Regarding the study presented in [18], only the satisfaction criterion was evaluated by comparing the system against face-to-face consultation. In [19], two types of evaluations were carried out: evaluative and formative, which focused on functionality and performance qualitative features with a group of 29 patients. Finally, the study presented in [20] describes a costs and savings evaluation about consultation time and hospital admissions of a telemedicine system integrated with video conference equipment that should be installed in the patients' homes.

Based on the review of studies presented by Wallace E.L, Rojas SV, and KS Nayak [15, 16, 21] and in this work, it is noted that there is limited research work focused on the assessment of the usability of telemonitoring systems through a case study with patients on CAPD/APD and medical staff in a real environment. Therefore, in our research, we included criteria that represent preponderant indicators that cannot be ignored, such as system visibility, congruence between the real world and the system, control and freedom, consistency and standards (organization), prevention of errors by an adequate design, recognition instead of memory, flexibility and efficiency of use, minimalist and aesthetic design, handling errors efficiently and help documentation, aim and purpose clarity (goals), navigability, interactivity, activities, and feedback. Likewise, criteria that are less frequent in the reviewed studies can be identified, and for that reason, it is interesting to explore them to make a contribution, such as motivation, satisfaction (recommendation) and applicability, which are criteria directly associated with the users' perspective. In this regard, this study considers intrapersonal aspects of the users subject to the stimulus generated by the system usage, giving an assessment oriented towards the system relevance for stakeholders, which allows for accurate feedback.

On the other hand, it is also important to establish a baseline for user satisfaction measures, with the aim of future system improvements that support adherence to the system and promote the recommendation of its usage to other stakeholders in PD therapies. Concerning the applicability criterion, it is necessary to know those system services that stakeholders may consider less relevant for the follow-up and control of their PD therapy, promoting systematic system updates.

Therefore, the usability analysis and assessment presented in this paper can serve as a reference point for system developers looking to improve or develop telemonitoring systems. It can also be seen that there is a need to standardize the usability assessment criteria for systems of this nature since, despite great efforts in the matter, system properties that motivate the creation of new evaluation criteria have emerged.

Based on the results obtained in the usability assessment, the system evaluated [13] in this study considers and satisfies the ideal requirements and parameters that should be monitored in PD treatment according to the study presented by KS Nayak [21].

## Conclusions

In this work, a usability assessment of a telemonitoring system for patients with CKD on PD treatment was presented based on a case study involving patients and doctors of the IMSS in a real environment. The results obtained from the case study show that, on average, the services offered to the patient by the system have 89.5% acceptance, with the APD and CAPD exchange data registration services being the most acceptable for patients, with a positive perception of 94.5% and 92.3%, respectively. On the other hand, services offered to medical staff obtained an average of 93.74% acceptance; in this case, the alarm reception service for patients in risk situations with 95% was the most accepted among the medical staff. Finally, it is noted that the evaluated system in this study considers and satisfies the requirements and suitable parameters that should be monitored in PD treatment according to the specialized literature.

## Supporting information

S1 File Instrument 1 (Spanish version). (PDF)

S2 File Instrument 2 (Spanish version). (PDF)

S3 File Instrument 1 (English version). (PDF)

S4 File Instrument 2 (English version). (PDF)
