# Supplementary material for: Telemonitoring system for patients with chronic kidney disease undergoing peritoneal dialysis: Usability assessment based on a case study
Source: PLoS One. 2018 Nov 6;13(11):e0206600. doi: 10.1371/journal.pone.0206600 (PMC6219778; doi:10.1371/journal.pone.0206600)
Supplement: S1 File — (PDF) [file pone.0206600.s001.pdf]

Este cuestionario tiene el objetivo de saber su perspectiva del sistema y satisfacción del uso de la misma. Las respuestas que usted nos proporcione tienen la finalidad de mejorar el monitoreo a distancia del tratamiento de los pacientes en diálisis peritoneal a través del sistema.

Cuestionario de: ____ Fecha: __________________

Usuario del sistema: ____________________________

Apartado de Recambio DPA/DPCA de la aplicación

1.- ¿En la opción de recambio de DPA, la aplicación le informa si está haciendo alguna operación?

En gran medida Moderadamente Poco Nada

2.- ¿El procedimiento del recambio DPA se ve reflejado en la opción de recambio DPA de la aplicación?

En gran medida Moderadamente Poco Nada

3.- ¿Toda la información que se solicita en su tratamiento de DPA se ingresa en la opción de recambio DPA de la aplicación?

En gran medida Moderadamente Poco Nada

4.- En caso de que las respuestas a las preguntas anteriores (2,3) fueran diferentes de "En gran medida" por favor describa el paso o la información que falta considerar:

5.- ¿Puede moverse libremente en la opción de recambio de DPA de la aplicación móvil?

En gran medida Moderadamente Poco Nada

6.- ¿Los elementos de la opción de recambio DPA de la aplicación móvil siempre aparecen en el mismo lugar?

En gran medida Moderadamente Poco Nada

7.- ¿El orden de los elementos de la opción de recambio DPA están organizados de acuerdo a cómo fueron explicados en la capacitación?

En gran medida Moderadamente Poco Nada

8.- ¿La forma en que se encuentra estructurada la información en la opción de recambio de DPA le permite ingresar fácilmente la información?

En gran medida Moderadamente Poco Nada

9.- ¿En la opción de recambio de DPA se le indica puntualmente el tipo de información que debe ingresar en los campos (Volumen de ingreso, tiempos a establecer, etc.)?

En gran medida Moderadamente Poco Nada

10.- ¿La presentación de la información en la opción de recambio de DPA de la aplicación lo guía adecuadamente a ingresar la información del tratamiento de DPA?

En gran medida Moderadamente Poco Nada

11.- ¿Es fácil identificar el objetivo de la opción de recambio de DPA?

En gran medida Moderadamente Poco Nada

12.- ¿La función que desempeñan todos los botones en la opción recambio de DPA es clara?

En gran medida Moderadamente Poco Nada

13.- ¿Es fácil distinguir los pasos que se deben llevar a cabo para realizar el ingreso de la información en la opción de recambio de DPA de la aplicación móvil?

En gran medida Moderadamente Poco Nada

14.- ¿Realizar el ingreso de la información en la opción de recambio de DPA de la aplicación móvil le lleva de 5 a 10 minutos?

En gran medida Moderadamente Poco Nada

15.- ¿La forma elegida para la presentación de la información (botones, campos de texto, etc.) en la opción de recambio de DPA de la aplicación es visualmente agradable?

En gran medida Moderadamente Poco Nada

16.- ¿El color de fondo en la opción de recambio de DPA de la aplicación es adecuado?

En gran medida Moderadamente Poco Nada

17.- ¿La letra del texto en la opción de recambio de DPA de la aplicación es legible?

En gran medida Moderadamente Poco Nada

18.- ¿Las imágenes y los colores de fondo de pantalla proporcionan suficiente contraste con el texto en la opción de recambio de DPA de la aplicación?

En gran medida Moderadamente Poco Nada

19.- ¿En la opción de recambio de DPA de la aplicación se informa adecuadamente si existe un error en el procedimiento?

En gran medida Moderadamente Poco Nada

20.- ¿El manejo de las opciones en el Registro de recambio de DPA siempre conduce a las páginas esperadas sin resultados no deseados (páginas inesperadas)?

En gran medida Moderadamente Poco Nada

21.- ¿Los mensajes proporcionados en la opción de recambio de DPA de la aplicación son claros?

En gran medida Moderadamente Poco Nada

22.- ¿El manual de usuario explica el procedimiento que se debe de llevar para la captura de la información del recambio de paciente con DPA?

En gran medida Moderadamente Poco Nada

23.- ¿Considera que la aplicación debería tener una opción de guía para resolver dudas y/o problemas?

En gran medida Moderadamente Poco Nada

24.- ¿La opción de recambio de DPA de la aplicación refleja correctamente el objetivo del tratamiento para pacientes con DPA?

En gran medida Moderadamente Poco Nada

25.- ¿Se idéntica claramente la información solicitada en la opción de recambio de DPA de la aplicación?

En gran medida Moderadamente Poco Nada

26.- ¿En la opción de recambio de DPA se ofrece ayuda de cómo navegar en sus elementos?

En gran medida Moderadamente Poco Nada

27.- ¿En la opción de recambio de DPA se permite retornar entre pantallas mediante un botón?

En gran medida Moderadamente Poco Nada

28.- ¿Al finalizar la captura de la información en la opción de recambio de DPA de la aplicación, se muestra un mensaje con la información guardada?

En gran medida Moderadamente Poco Nada

29.- ¿En la opción de recambio de DPA de la aplicación se informa si ya se ha capturado el recambio del día?

En gran medida Moderadamente Poco Nada

30.- ¿En la opción de recambio de DPA de la aplicación se informa cuando se genera una alerta?

En gran medida Moderadamente Poco Nada

31.- ¿Considera que los datos ingresados en la opción de recambio de la aplicación son adecuados para informar al médico(a)/enfermero(a) del tratamiento de DPA del paciente?

En gran medida Moderadamente Poco Nada

32.- ¿Considera que la opción de recambio de DPA de la aplicación ha mejorado el monitoreo de su tratamiento DPA?

En gran medida Moderadamente Poco Nada

33.- ¿El uso de la opción de recambio de DPA de la aplicación le ha facilitado la captura y control de los datos generados por el tratamiento de DPA en comparación con el método tradicional (captura en el formato de papel impreso)?

En gran medida Moderadamente Poco Nada

34.- ¿La interacción médico-paciente resultante de la opción de recambio de DPA de la aplicación lo motiva a seguir utilizándola?

En gran medida Moderadamente Poco Nada

35.- ¿Recomendaría la opción de recambio de DPA de la aplicación para que la utilizarán otros pacientes para captura y monitoreo de su tratamiento de DPA?

En gran medida Moderadamente Poco Nada

36.- ¿Considera más agradable y fácil el uso de la opción de recambio de DPA de la aplicación en comparación al método tradicional (captura en el formato de papel impreso)?

En gran medida Moderadamente Poco Nada

37.- ¿Interactuar con la opción de recambio de DPA de la aplicación le ha sido de utilidad para la captura y monitoreo de su tratamiento?

En gran medida Moderadamente Poco Nada

38.- ¿Considera que la opción de recambio de DPA de la aplicación es un complemento al monitoreo de su tratamiento de DPA?

En gran medida Moderadamente Poco Nada

39.- Nos podría proporcionar por favor alguna recomendación o comentario para mejorar la opción de recambio de DPA de la aplicación móvil:

Alertas o avisos de la aplicación

1.- ¿La aplicación le informa si está enviando alguna alerta o aviso a médicos y enfermeras?

En gran medida Moderadamente Poco Nada

2.- ¿Las alertas generadas son creadas en el momento adecuado (cuando el líquido de salida no es transparente, ultrafiltración negativa, etc.)?

En gran medida Moderadamente Poco Nada

3.- ¿Las alertas siempre se informan en las mismas pantallas?

En gran medida Moderadamente Poco Nada

4.- ¿Las alertas generadas siempre se muestran en la misma posición?

En gran medida Moderadamente Poco Nada

5.- ¿La información de la alerta siempre indica la razón por la cual se generó?

En gran medida Moderadamente Poco Nada

6.- ¿En las opciones de recambio, las alertas se generan de forma inmediata?

En gran medida Moderadamente Poco Nada

7.- ¿Las alertas generadas en la aplicación son claras?

En gran medida Moderadamente Poco Nada

8.- ¿El objetivo de la generación de alertas en la aplicación es claro?

En gran medida Moderadamente Poco Nada

9.- ¿En la opción de recambio se informa cuando se genera una alerta?

En gran medida Moderadamente Poco Nada

10.- ¿Las alertas generadas tienen respuesta por parte del médico mediante una notificación?

En gran medida Moderadamente Poco Nada

11.- ¿Considera que las alertas son parte fundamental de la comunicación con los médicos(as)/Enfermeros(as)?

En gran medida   Moderadamente   Poco   Nada

12.- ¿Considera que las alertas generadas de la aplicación es un complemento para el monitoreo del tratamiento?

En gran medida   Moderadamente   Poco   Nada

13.- ¿Nos podría proporcionar alguna recomendación para mejorar la opción de Generación de Alertas o Avisos de la aplicación:

14.- De las diferentes opciones de la aplicación cuál considera de mayor importancia y que recomendaciones generales nos propone para mejorar la aplicación

Apartado de Notificaciones de la aplicación

1.- ¿En la opción de Notificaciones la aplicación le informa si está haciendo alguna operación?

En gran medida Moderadamente Poco Nada

2.- ¿El procedimiento de retroalimentación (recomendaciones, recordatorios, citas, etc) por parte del médico se ve reflejado en la opción de notificaciones de la aplicación?

En gran medida Moderadamente Poco Nada

3.- ¿Los tipos de notificaciones presentadas en la aplicación son las únicas que recibe?

En gran medida Moderadamente Poco Nada

4.- En caso de que las respuestas a las preguntas anteriores (2,3) fueran diferentes de "En gran medida" por favor describa porque no se refleja las notificaciones o qué tipo de notificación desearía que se mostrará:

5.- ¿Puede moverse libremente en la opción de Notificaciones en la aplicación?

En gran medida Moderadamente Poco Nada

6.- ¿Los elementos (botones, etiquetas, etc) de las notificaciones siempre aparecen en el mismo lugar?

En gran medida Moderadamente Poco Nada

7.- ¿Los elementos de las notificaciones (botones, etiquetas, etc) están organizados de acuerdo a lo explicado en la guía de usuario de la aplicación?

En gran medida Moderadamente Poco Nada

8.- ¿La forma en que se encuentra estructurada la información en la opción de notificaciones ayuda a identificar el tipo de notificación?

En gran medida Moderadamente Poco Nada

9.- ¿En la opción de Notificaciones la aplicación indica puntualmente el tipo de notificaciones que ha recibido?

En gran medida Moderadamente Poco Nada

10.- ¿El orden en la opción de notificaciones lo guía adecuadamente para ver las diferentes notificaciones?

En gran medida Moderadamente Poco Nada

11.- ¿Es fácil distinguir en la opción de notificaciones el objetivo de cada notificación?

En gran medida Moderadamente Poco Nada

12.- ¿La función que desempeñan todos los botones en la opción de notificaciones es clara?

En gran medida Moderadamente Poco Nada

13.- ¿Es fácil distinguir los pasos que se deben llevar a cabo para visualizar las diferentes notificaciones?

En gran medida Moderadamente Poco Nada

14.- ¿La forma elegida para la presentación de las notificaciones que usted recibe por parte del personal médico y de enfermería es visualmente agradable?

En gran medida Moderadamente Poco Nada

15.- ¿El color de fondo en la opción de notificaciones de la aplicación es adecuado?

En gran medida Moderadamente Poco Nada

16.- ¿La letra del texto en la opción de notificaciones de la aplicación es fácil de leer?

En gran medida Moderadamente Poco Nada

17.- ¿Las imágenes y los colores de fondo de pantalla proporcionan suficiente contraste con el texto en la opción de notificaciones de la aplicación?

En gran medida Moderadamente Poco Nada

18.- ¿En la opción de notificaciones de la aplicación se informa adecuadamente si existe un error en el procedimiento?

En gran medida Moderadamente Poco Nada

20.- ¿Los mensajes proporcionados en la opción de notificaciones de la aplicación son claros?

En gran medida Moderadamente Poco Nada

21.- ¿El manual de usuario explica el procedimiento que se debe de llevar a cabo para la revisión de las notificaciones en la aplicación?

En gran medida Moderadamente Poco Nada

22.- ¿Considera que la aplicación debería tener una opción de guía para resolver dudas y/o problemas sobre la consulta de las notificaciones?

En gran medida Moderadamente Poco Nada

23.- ¿La aplicación refleja correctamente el objetivo de las notificaciones?

En gran medida Moderadamente Poco Nada

24.- ¿En el tipo de notificaciones de la aplicación su propósito es claro?

En gran medida Moderadamente Poco Nada

25.- ¿En la opción de notificaciones de la aplicación se ofrece ayuda de cómo navegar en sus elementos?

En gran medida Moderadamente Poco Nada

26.- ¿En la opción de notificaciones se permite retornar entre pantallas mediante un botón y/o utilizando el botón de regreso del teléfono?

En gran medida Moderadamente Poco Nada

27.- ¿La aplicación le informa cuando se recibió una nueva notificación?

En gran medida Moderadamente Poco Nada

28.- ¿En la opción de notificaciones se informa el estatus de las notificaciones (nueva, leída)?

En gran medida Moderadamente Poco Nada

29.- ¿Considera que las notificaciones en la aplicación son resultado del seguimiento adecuado del médico(a)/enfermero(a) sobre tratamiento de DPCA del paciente?

En gran medida Moderadamente Poco Nada

30.- ¿Considera que la opción de notificaciones de la aplicación ha mejorado el monitoreo de su tratamiento DPCA?

En gran medida Moderadamente Poco Nada

31.- ¿El uso de la opción de notificaciones de la aplicación le ha facilitado la comunicación con el médico(a)/enfermero(a) sobre el tratamiento de DPCA?

En gran medida Moderadamente Poco Nada

19.-¿El manejo de las opciones dentro de la sección de notificaciones siempre conduce a las páginas esperadas sin resultados no deseados (páginas inesperadas)?

En gran medida Moderadamente Poco Nada

20.-¿El manejo de las opciones en el Registro de recambio de DPCA siempre conduce a las páginas esperadas sin resultados no deseados (páginas inesperadas)?

En gran medida Moderadamente Poco Nada

32.- ¿Recomendaría la opción de notificaciones de la aplicación para que la utilizarán otros pacientes?

En gran medida Moderadamente Poco Nada

33.- ¿Considera más agradable y fácil el uso de la opción de notificaciones para mantenerse en contacto con los médicos(as)/Enfermeros(as) qué por otros medios de comunicación?

En gran medida Moderadamente Poco Nada

34.- ¿Considera que la opción de notificaciones de la aplicación complementa el monitoreo de su tratamiento por parte del personal médico y de enfermería?

En gran medida Moderadamente Poco Nada

35.- ¿Nos podría proporcionar alguna recomendación para mejorar la opción de Notificaciones de la aplicación móvil:
