# Supplementary material for: Telemonitoring system for patients with chronic kidney disease undergoing peritoneal dialysis: Usability assessment based on a case study
Source: PLoS One. 2018 Nov 6;13(11):e0206600. doi: 10.1371/journal.pone.0206600 (PMC6219778; doi:10.1371/journal.pone.0206600)
Supplement: S2 File — (PDF) [file pone.0206600.s002.pdf]

Este cuestionario tiene el objetivo de saber su perspectiva del sistema y satisfacción del uso de la misma. Las respuestas que usted nos proporcione tienen la finalidad de mejorar el monitoreo a distancia del tratamiento de los pacientes en diálisis peritoneal a través del sistema.

Cuestionario de: ____ Fecha: __________________

Usuario del sistema:____________________________

I: Cuestionario de Registro de Diálisis

1. ¿En las opciones de Registro de diálisis al momento de realizar la consulta del resumen detallado o diario del paciente, el sistema le informa si está haciendo alguna operación, p.e. cargando datos?

En gran medida Moderadamente Poco Nada

2. ¿Toda la información que se presenta en la opción de registros de diálisis corresponde a los datos necesarios para darle seguimiento al tratamiento de su paciente?

En gran medida Moderadamente Poco Nada

3. ¿Puede moverse libremente en las opciones de Registro de diálisis en el sistema?

En gran medida Moderadamente Poco Nada

4. ¿Los elementos de fechas, resumen detallado, resumen diario, etc. en la opción de Registro de diálisis siempre aparecen en el mismo lugar?

En gran medida Moderadamente Poco Nada

5. ¿La representación de la información del resumen detallado o diario que se muestra en la opción de Registro de diálisis te permite analizar adecuadamente el balance general y/o ultrafiltración?

En gran medida Moderadamente Poco Nada

6. ¿En la opción de Registro de diálisis del sistema se indica puntualmente el tipo de información que debe ingresar en las fechas de la consulta?

En gran medida Moderadamente Poco Nada

7. ¿El sistema lo guía adecuadamente para consultar la información requerida en la opción de Registro de diálisis?

En gran medida Moderadamente Poco Nada

8. ¿Es fácil distinguir el objetivo de la opción de Registro de diálisis del sistema?

En gran medida Moderadamente Poco Nada

9. ¿La función que desempeñan todos los botones en la opción de Registro de diálisis es clara?

En gran medida Moderadamente Poco Nada

10. ¿Realizar la consulta de información en la opción de Registro de diálisis le lleva de 2 a 5 minutos?

En gran medida Moderadamente Poco Nada

11. ¿En la opción de Registro de diálisis es visualmente agradable la forma elegida para la presentación de la información del paciente?

En gran medida Moderadamente Poco Nada

12. ¿El color de fondo en la opción de Registro de diálisis del sistema es adecuado?

En gran medida Moderadamente Poco Nada

13. ¿La letra del texto en la opción de Registro de diálisis del sistema es legible?

En gran medida Moderadamente Poco Nada

14. ¿En la opción de Registro de diálisis del sistema, las imágenes y los colores de fondo de pantalla proporcionan suficiente contraste con el texto?

En gran medida Moderadamente Poco Nada

15. ¿En la opción de Registro de diálisis del sistema se le informa adecuadamente si existe un error en la consulta de información?

En gran medida Moderadamente Poco Nada

16. ¿El manejo de las opciones en el Registro de diálisis del sistema siempre conduce a las páginas esperadas sin resultados no deseados (páginas inesperadas)?

En gran medida Moderadamente Poco Nada

17. ¿En la opción de Registro de diálisis, los mensajes de aviso, confirmación y error son claros?

En gran medida Moderadamente Poco Nada

18. ¿El manual de usuario le explica el procedimiento que se debe de llevar a cabo para la consulta de la información del paciente en la opción de Registros de diálisis del sistema?

En gran medida Moderadamente Poco Nada

19. ¿Considera que el sistema debería tener una opción de guía para resolver dudas y/o problemas?

En gran medida Moderadamente Poco Nada

20. ¿La información presentada en la opción de Registros de diálisis refleja correctamente el seguimiento del tratamiento del paciente?

En gran medida Moderadamente Poco Nada

21. ¿La información que se le presenta en la opción de Registros de diálisis del sistema es clara?

En gran medida Moderadamente Poco Nada

22. ¿En la opción de Registros de diálisis del sistema se le ofrece ayuda de cómo navegar en sus elementos?

En gran medida Moderadamente Poco Nada

23. ¿En la opción de Registros de diálisis del sistema se le permite retornar entre pantallas mediante un botón?

En gran medida Moderadamente Poco Nada

24. ¿En la opción de Registros de diálisis la información presentada corresponde a los periodos de tiempo solicitados?

En gran medida Moderadamente Poco Nada

25. ¿Los pasos para realizar las consultas en la opción Registros de diálisis son claros?

En gran medida Moderadamente Poco Nada

26. ¿Considera que la información presentada en la opción de Registros de diálisis del sistema es suficiente para el monitoreo del tratamiento del paciente?

En gran medida Moderadamente Poco Nada

27. ¿Considera que los datos presentados en la opción de registro de diálisis del sistema son adecuados para informar al médico(a)/enfermero(a) del tratamiento su paciente?

En gran medida Moderadamente Poco Nada

28. ¿Considera que las opciones de Registro de diálisis del sistema han mejorado el monitoreo del tratamiento de sus pacientes?

En gran medida Moderadamente Poco Nada

29. ¿El uso de la opción de Registro de diálisis del sistema le ha facilitado el acceso a la información del tratamiento de tu paciente?

En gran medida Moderadamente Poco Nada

30. ¿La interacción médico-paciente resultante de la opción de Registro de diálisis lo motiva a seguir utilizando el sistema?

En gran medida Moderadamente Poco Nada

31. ¿Recomendaría la opción de Registro de diálisis del sistema para que la utilizarán otros médicos para el monitoreo de sus pacientes de DPA/DPCA?

En gran medida Moderadamente Poco Nada

32. ¿Considera más agradable y fácil la consulta de la información del tratamiento de su paciente en comparación al método tradicional (registro en papel por parte del paciente)?

En gran medida Moderadamente Poco Nada

33. ¿Interactuar con la opción de Registro de diálisis del sistema le ha sido de utilidad para el monitoreo a distancia de tu paciente?

En gran medida Moderadamente Poco Nada

34. ¿Considera que la opción de Registro de diálisis del sistema es un complemento en el monitoreo del tratamiento de sus pacientes en DPA/DPCA?

En gran medida Moderadamente Poco Nada

35. ¿Considera que la opción de Registro de diálisis del sistema ha contribuido a llevar a cabo un monitoreo del paciente más efectivo/puntual?

En gran medida Moderadamente Poco Nada

36. ¿Nos podría proporcionar alguna recomendación para mejorar la opción de Registro de diálisis del sistema?

II: Cuestionario de Alertas o avisos

1. ¿Al momento de consultar las alertas generadas por los pacientes en la opción de revisar alertas, el sistema le informa si está haciendo alguna operación?

En gran medida Moderadamente Poco Nada

2. ¿En la opción de revisar alertas la información presentada es congruente para el seguimiento del tratamiento del paciente?

En gran medida Moderadamente Poco Nada

3. ¿Toda la información que se presenta en la opción de revisar alertas corresponde a la solicitada?

En gran medida Moderadamente Poco Nada

4. ¿Puede moverse libremente en la opción de revisar alertas del sistema?

En gran medida Moderadamente Poco Nada

5. ¿Los elementos de fechas, historial, gráficas, etc. de revisar alertas siempre aparecen en el mismo lugar?

En gran medida Moderadamente Poco Nada

6. ¿La estructura presentada en el reporte de alertas y en la gráfica le permite analizar el comportamiento del tratamiento del paciente?

En gran medida Moderadamente Poco Nada

7. ¿En la opción de revisar alertas del sistema se indica puntualmente el formato de las fechas que se requiere para realizar la consulta?

En gran medida Moderadamente Poco Nada

8. ¿Es fácil distinguir el objetivo de la opción de revisar alertar del sistema?

En gran medida Moderadamente Poco Nada

9. ¿La función que desempeñan todos los botones en la opción de revisar alertas es clara?

En gran medida Moderadamente Poco Nada

10. ¿Realizar la consulta de alertas te lleva entre 2 y 5 minutos?

En gran medida Moderadamente Poco Nada

11. ¿En la opción revisar alertas es visualmente agradable la forma elegida para la representación de la información del tratamiento del paciente?

En gran medida Moderadamente Poco Nada

12. ¿El color de fondo en la opción de revisar alertas del sistema es adecuado?

En gran medida Moderadamente Poco Nada

13. ¿La letra del texto en la opción de revisar alertas del sistema es legible?

En gran medida Moderadamente Poco Nada

14. ¿En la opción de revisar alertas del sistema, las imágenes y los colores de fondo de pantalla proporcionan suficiente contraste con el texto?

En gran medida Moderadamente Poco Nada

15. ¿En la opción de revisar alertas del sistema se informa adecuadamente si existe un error al solicitar el reporte de alertas?

En gran medida Moderadamente Poco Nada

16. ¿El manejo de las opciones de revisar alertas del sistema siempre conduce a las páginas esperadas sin resultados no deseados (páginas inesperadas)?

En gran medida Moderadamente Poco Nada

17. ¿El manual de usuario explica el procedimiento que se debe de llevar a cabo para la consulta de alertas de su paciente en la opción de revisar alertas del sistema?

En gran medida Moderadamente Poco Nada

18. ¿Considera que el sistema debería tener una opción de guía para resolver dudas y/o problemas sobre la consulta de las alertas?

En gran medida Moderadamente Poco Nada

19. ¿El objetivo de la opción de revisar alertas del sistema se refleja correctamente?

En gran medida Moderadamente Poco Nada

20. ¿En la opción de revisar alertas del sistema, la información que se le presenta es clara?

En gran medida Moderadamente Poco Nada

21. ¿En la opción de revisar alertas del sistema se ofrece ayuda de cómo navegar en sus elementos?

En gran medida Moderadamente Poco Nada

22. ¿En la opción de revisar alertas, el sistema le permite retornar entre pantallas mediante un botón?

En gran medida Moderadamente Poco Nada

23. ¿Los pasos para hacer una consulta en la opción de revisar alertas son claros?

En gran medida Moderadamente Poco Nada

24. ¿Las alertas recibidas (SMS o correo) te proporcionan información suficiente ya sea del tratamiento (características del líquido y ultrafiltración) o de sus rangos de datos médicos?

En gran medida Moderadamente Poco Nada

25. ¿Considera que los datos presentados en la opción de revisar alertas del sistema son adecuados para informar al médico(a)/enfermero(a) del tratamiento del paciente?

En gran medida Moderadamente Poco Nada

26. ¿Considera que la opción de revisar alertas del sistema ha mejorado el monitoreo del tratamiento de sus pacientes?

En gran medida Moderadamente Poco Nada

27. ¿El uso de la opción de revisar alertas del sistema le ha facilitado el control de los datos generados por el tratamiento del paciente?

En gran medida Moderadamente Poco Nada

28. ¿La interacción médico-paciente resultante de la opción de revisar alertas del sistema lo motiva a llevar a cabo una comunicación precisa con sus pacientes?

En gran medida Moderadamente Poco Nada

29. ¿Recomendaría la opción de revisar alertas del sistema para que la utilizarán otros médicos en el control de sus pacientes en DPA/DPCA?

En gran medida Moderadamente Poco Nada

30. ¿Considera más eficiente el uso de la opción de revisar alertas del sistema a esperar a que los pacientes reporten alguna condición de su tratamiento?

En gran medida Moderadamente Poco Nada

31. ¿Interactuar con la opción de revisar alertas del sistema le ha sido de utilidad para el monitoreo de su paciente?

En gran medida Moderadamente Poco Nada

32. ¿Interactuar con la opción de revisar alertas del sistema le ha sido de utilidad para informar al paciente de la acción a realizar?

En gran medida Moderadamente Poco Nada

33. ¿Considera que la opción de revisar alertas del sistema es un complemento del monitoreo del tratamiento de los pacientes?

En gran medida Moderadamente Poco Nada

34. ¿Nos podría proporcionar alguna recomendación para mejorar la opción de revisar alertas del sistema?

III: Cuestionario de Notificaciones

1. ¿En la opción de generar notificaciones, el sistema le informa del estado de la notificación (no sincronizada, entregada, etc)?

En gran medida Moderadamente Poco Nada

2. ¿La generación de notificaciones (recomendaciones, recordatorios, citas, etc.) permite al médico brindar retroalimentación al paciente de su tratamiento?

En gran medida Moderadamente Poco Nada

3. ¿Usted considera que la información que se agrega en las notificaciones es de utilidad para mantener informado al paciente sobre su tratamiento?

En gran medida Moderadamente Poco Nada

4. ¿Considera adecuado el procedimiento de generar notificaciones para informar al paciente sobre su tratamiento?

En gran medida Moderadamente Poco Nada

5. En caso de que las respuestas a las preguntas anteriores (3,4) fueran diferentes de "En gran medida" por favor describa el procedimiento o la información que sería deseable considerar:

6. ¿Puede moverse libremente en la opción de generar notificaciones en el sistema?

En gran medida Moderadamente Poco Nada

7. ¿Las notificaciones generadas para el tratamiento del paciente corresponden a las que se presentan en la pantalla general de la opción de generar notificaciones?

En gran medida Moderadamente Poco Nada

8. ¿La información que se ingresa en la opción de generar notificaciones es adecuada para orientar al paciente sobre su tratamiento?

En gran medida Moderadamente Poco Nada

9. ¿En el apartado de generar notificaciones se le indica puntualmente el tipo de información que debe ingresar en los campos (Tipo de notificación, descripción y fecha de expiración, etc.)?

En gran medida Moderadamente Poco Nada

10. ¿Es fácil distinguir el objetivo de la opción de generar notificaciones del sistema?

En gran medida Moderadamente Poco Nada

11. ¿El reporte de notificaciones le permite distinguir las diferentes notificaciones que se han realizado al paciente?

En gran medida Moderadamente Poco Nada

12. ¿El sistema le permite el registro de notificaciones de manera intuitiva?

En gran medida Moderadamente Poco Nada

13. ¿El procedimiento para realizar cualquier tipo de notificaciones es similar en los diferentes tipos de notificación?

En gran medida Moderadamente Poco Nada

14. ¿Realizar una notificación para un paciente en el sistema le lleva de 2 a 5 minutos?

En gran medida Moderadamente Poco Nada

15. ¿En el apartado de generar notificaciones, el sistema le permite cancelar o realizar los cambios requeridos?

En gran medida Moderadamente Poco Nada

16. ¿En la opción de generar de notificaciones del sistema es visualmente agradable la forma elegida para la captura y presentación de la información?

En gran medida Moderadamente Poco Nada

17. ¿El color de fondo en la opción de generación de notificaciones es adecuado?

En gran medida Moderadamente Poco Nada

18. ¿La letra del texto en la opción de generación de notificaciones es legible?

En gran medida Moderadamente Poco Nada

19. ¿En la opción generar notificaciones las imágenes y los colores de fondo de pantalla proporcionan suficiente contraste con el texto?

En gran medida Moderadamente Poco Nada

20. ¿En el caso de haberse quedado sin acceso a internet(wifi), el sistema le informa de dicho estado?

En gran medida Moderadamente Poco Nada

21. ¿El manejo de las opciones de generar notificaciones del sistema siempre conduce a las páginas esperadas sin resultados no deseados (páginas inesperadas)?

En gran medida Moderadamente Poco Nada

22. ¿El manual de usuario explica el procedimiento que se debe de llevar a cabo para la consulta y/o captura de notificaciones?

En gran medida Moderadamente Poco Nada

23. ¿La información solicitada en el apartado de notificaciones refleja correctamente el objetivo de dicho servicio?

En gran medida Moderadamente Poco Nada

24. ¿En la opción de generar notificaciones del sistema se ofrece ayuda de cómo navegar en sus elementos?

En gran medida Moderadamente Poco Nada

25. ¿En la opción de generar notificaciones del sistema se permite retornar o moverse entre pantallas mediante un botón?

En gran medida Moderadamente Poco Nada

26. ¿Las notificaciones generadas se reflejan de manera adecuada y oportuna en la tabla de notificaciones?

En gran medida Moderadamente Poco Nada

27. ¿En la opción de generar notificaciones se distingue el estado de las notificaciones?

En gran medida Moderadamente Poco Nada

28. ¿Considera que la opción de generar notificaciones del sistema ha mejorado la comunicación con sus pacientes?

En gran medida Moderadamente Poco Nada

29. ¿El uso de la opción de generar notificaciones le ha facilitado el seguimiento y control de los pacientes?

En gran medida Moderadamente Poco Nada

30. ¿Recomendarías la opción de generar notificaciones para que la utilizarán otros médicos para el seguimiento y control de los pacientes?

En gran medida Moderadamente Poco Nada

31. ¿Interactuar con la opción de generar notificaciones le ha sido de utilidad en la comunicación de sus pacientes?

En gran medida Moderadamente Poco Nada

32. ¿Considera que la opción de generar notificaciones es un complemento para el seguimiento y control de los pacientes en tratamiento de DPA/DPCA?

En gran medida Moderadamente Poco Nada

33. ¿Nos podría proporcionar alguna recomendación para mejorar la opción de generar notificaciones del sistema?

34. De las diferentes opciones del sistema cuál considera de mayor importancia y que recomendaciones generales nos propone para mejorar el sistema:
