# Supplementary material for: Telemonitoring system for patients with chronic kidney disease undergoing peritoneal dialysis: Usability assessment based on a case study
Source: PLoS One. 2018 Nov 6;13(11):e0206600. doi: 10.1371/journal.pone.0206600 (PMC6219778; doi:10.1371/journal.pone.0206600)
Supplement: S3 File — (PDF) [file pone.0206600.s003.pdf]

This questionnaire has the objective of knowing your perspective about the system, as well as the satisfaction of use. The answers you provide will help us to improve the remote monitoring of the treatment of patients on peritoneal dialysis through the system.

Questionnaire: _______________________________________ Date: __________________

System user: ____________________________

Exchange APD/CAPD section of the application

1.- In the APD exchange option, the application informs you if it is doing any operation?

Strongly agree Moderately Agree Somewhat agree Neutral

2.- Is the APD exchange procedure reflected in the APD exchange application option?

Strongly agree Moderately Agree Somewhat agree Neutral

3.- Is all the information requested in your APD treatment entered in the APD exchange option of the application?

Strongly agree Moderately Agree Somewhat agree Neutral

4.- In case answers to the previous questions (2,3) were different from "Strongly agree" please describe the action or the information that needs to be considered:

5.- Can you browse freely in the APD exchange option of the mobile application?

Strongly agree Moderately Agree Somewhat agree Neutral

6.- Do the elements of the APD exchange option of the mobile application always appear in the same place?

Strongly agree Moderately Agree Somewhat agree Neutral

7.- Is the order of the elements in the APD exchange option organized according to the form they were explained during the training?

Strongly agree Moderately Agree Somewhat agree Neutral

8.- The way in which the information is structured within the APD exchange option allows you to easily enter the information?

Strongly agree Moderately Agree Somewhat agree Neutral

9.- In the APD exchange option, is explicitly indicated the type of information that must be entered in the fields (volume, time to establish, etc.)?

Strongly agree Moderately Agree Somewhat agree Neutral

10.- Does the presentation of the information in the APD exchange application option guide you appropriately to enter the APD treatment information?

Strongly agree Moderately Agree Somewhat agree Neutral

11.- Is it easy to identify the purpose of the APD exchange option?

Strongly agree Moderately Agree Somewhat agree Neutral

12.- Is the function played by all the buttons within the APD exchange option clear?

Strongly agree Moderately Agree Somewhat agree Neutral

13.- Is it easy to distinguish the steps that must be carried out to perform the entry of the information in the APD exchange option of the mobile application?

Strongly agree Moderately Agree Somewhat agree Neutral

14.- Do the entry of the information within the APD exchange option of the mobile application takes 5 to 10 minutes?

Strongly agree Moderately Agree Somewhat agree Neutral

15.- Does the form chosen for the information presentation (buttons, text fields, etc.) within the APD exchange option of the application is visually pleasing?

Strongly agree Moderately Agree Somewhat agree Neutral

16.- Is the background color in the APD exchange option of the application adequate?

Strongly agree Moderately Agree Somewhat agree Neutral

17.- Is the text readable in the APD exchange option of the application?

Strongly agree Moderately Agree Somewhat agree Neutral

18.- Do images and background colors provide enough contrast with the text in the APD exchange option of the application?

Strongly agree   Moderately Agree   Somewhat agree   Neutral

19.- Does the APD exchange option of the application, inform you properly if there is an error in the procedure?

Strongly agree   Moderately Agree   Somewhat agree   Neutral

20.- Does the APD exchange record option management always lead to the expected pages without unwanted results (unexpected pages)?

Strongly agree   Moderately Agree   Somewhat agree   Neutral

21.- Are the messages provided in the APD exchange option of the application clear?

Strongly agree   Moderately Agree   Somewhat agree   Neutral

22.- Does the user manual explain the procedure that must be followed to capture the exchange information of patients with APD?

Strongly agree   Moderately Agree   Somewhat agree   Neutral

23.- Do you think that the application should have a guide option to solve doubts and/or problems?

Strongly agree   Moderately Agree   Somewhat agree   Neutral

24.- Does the APD exchange option of the application correctly reflect the goal of treatment for patients with APD?

Strongly agree   Moderately Agree   Somewhat agree   Neutral

25.- Is the information requested in the APD exchange option of the application clearly identified?

Strongly agree   Moderately Agree   Somewhat agree   Neutral

26.- Does the APD exchange option offer help about how to navigate through its elements?

Strongly agree   Moderately Agree   Somewhat agree   Neutral

27.- In the APD exchange option is it possible to return between screens using a button?

Strongly agree Moderately Agree Somewhat agree Neutral

28.- When the capture of the information in the APD exchange option of the application finishes, is a message with the saved information displayed?

Strongly agree Moderately Agree Somewhat agree Neutral

29.- In the APD exchange option of the application is it informed if the exchange of the day has already been captured?

Strongly agree Moderately Agree Somewhat agree Neutral

30.- Is the APD exchange option of the application informed when an alert is generated?

Strongly agree Moderately Agree Somewhat agree Neutral

31.- Do you consider that the data entered in the exchange option of the application are adequate to inform the doctor/nurse of the patient's APD treatment?

Strongly agree Moderately Agree Somewhat agree Neutral

32.- Do you think that the application's APD exchange option has improved the monitoring of your APD treatment?

Strongly agree Moderately Agree Somewhat agree Neutral

33.- Has the use of the application's APD exchange option made it easier for you to capture and control the data generated by the APD treatment compared to the traditional method (captured in a printed format)?

Strongly agree Moderately Agree Somewhat agree Neutral

34.- Does the patient-physician interaction resulting from the application's APD exchange option motivate you to continue using it?

Strongly agree Moderately Agree Somewhat agree Neutral

35.- Would you recommend the application's APD exchange option to other patients for capturing and monitoring their APD treatment?

Strongly agree Moderately Agree Somewhat agree Neutral

36.- Do you consider more pleasant and easy to use the application's APD exchange option compared to the traditional method (captured in a printed format)?

Strongly agree Moderately Agree Somewhat agree Neutral

37.- Interacting with the APD exchange option of the application has been useful for capturing and monitoring your treatment?

Strongly agree Moderately Agree Somewhat agree Neutral

38.- Do you consider that the application's APD exchange option is a complement to the monitoring of your APD treatment?

Strongly agree Moderately Agree Somewhat agree Neutral

39.- Could you please provide us with any recommendation or comment to improve the APD exchange option of the mobile application:

Application's Alerts or Notifications

1.-Does the application notify you when an alert or warning is sent to doctors and nurses?

Strongly agree   Moderately Agree   Somewhat agree   Neutral

2.- Are the generated alerts created at the right time (when the drained fluid is not transparent, negative ultrafiltration, etc.)?

Strongly agree   Moderately Agree   Somewhat agree   Neutral

3.- Are alerts always reported on the same screens?

Strongly agree   Moderately Agree   Somewhat agree   Neutral

4.- Are the generated alerts always displayed in the same position?

Strongly agree   Moderately Agree   Somewhat agree   Neutral

5.- Does the alert information always indicate the reason it was generated?

Strongly agree   Moderately Agree   Somewhat agree   Neutral

6.- In exchange options, alerts are generated immediately?

Strongly agree   Moderately Agree   Somewhat agree   Neutral

7.- Are the generated alerts in the application clear?

Strongly agree   Moderately Agree   Somewhat agree   Neutral

8.- Is the purpose of generating alerts in the application clear?

Strongly agree   Moderately Agree   Somewhat agree   Neutral

9.- Is the exchange option informed when an alert is generated?

Strongly agree   Moderately Agree   Somewhat agree   Neutral

10.- Are the alerts generated answered by the doctor through a notification?

Strongly agree   Moderately Agree   Somewhat agree   Neutral

11.- Do you consider that alerts are an essential part of communication with doctors/nurses?

Strongly agree Moderately Agree Somewhat agree Neutral

12.- Do you think that alerts generated from the application are a complement for monitoring the treatment?

Strongly agree Moderately Agree Somewhat agree Neutral

13.- Could you provide us with any recommendation to improve the option of application's Alerts or Notices generation:

14.- Regarding the different application's options, which one do you consider most important and which general recommendations do you propose to improve the application?

Application's Notifications Section

1.- In the Notifications option, the application informs you if it is doing any operation?

Strongly agree Moderately Agree Somewhat agree Neutral

2.- Is the feedback procedure (recommendations, reminders, appointments, etc.) by the doctor reflected in the application's notification option?

Strongly agree Moderately Agree Somewhat agree Neutral

3.- Are the types of notifications presented in the application the only ones you receive?

Strongly agree Moderately Agree Somewhat agree Neutral

4.- In case the answers to the previous questions (2,3) were different from "Strongly agree" please describe why the notifications are not reflected or what kind of notification you would like to be displayed:

5.- Can you browse freely within the Notifications option of the application?

Strongly agree Moderately Agree Somewhat agree Neutral

6.- Do the elements (buttons, labels, etc.) of the notifications always appear in the same place?

Strongly agree Moderately Agree Somewhat agree Neutral

7.- Are the elements of the notifications (buttons, labels, etc.) organized according to what is explained in the application's user guide?

Strongly agree Moderately Agree Somewhat agree Neutral

8.- Does the structured information within the notification option help you to identify the type of notification?

Strongly agree Moderately Agree Somewhat agree Neutral

9.- In the Notifications option, does the application specifically indicate the type of notifications that you have received?

Strongly agree Moderately Agree Somewhat agree Neutral

10.- Does the order in the notifications option guide you properly to see the different notifications?

Strongly agree Moderately Agree Somewhat agree Neutral

11.- Is it easy to distinguish in the notification option the purpose of each notification?

Strongly agree Moderately Agree Somewhat agree Neutral

12.- Is the function performed by all the buttons in the notification option clear?

Strongly agree Moderately Agree Somewhat agree Neutral

13.- Is it easy to distinguish the actions that must be carried out to visualize the different notifications?

Strongly agree Moderately Agree Somewhat agree Neutral

14.- Is the form chosen for the notifications' presentation that you receive from the medical and nursing staff visually pleasing?

Strongly agree Moderately Agree Somewhat agree Neutral

15.- Is the background color adequate in the application's notification option?

Strongly agree Moderately Agree Somewhat agree Neutral

16.- Is the text in the application's notification option easy to read?

Strongly agree Moderately Agree Somewhat agree Neutral

17.- Do images and background colors provide enough contrast with the text in the application notifications option?

Strongly agree Moderately Agree Somewhat agree Neutral

18.- Does the notification option of the application notify you properly if there is an error in the procedure?

Strongly agree Moderately Agree Somewhat agree Neutral

20.- Are the messages provided in the application's notification option clear?

Strongly agree Moderately Agree Somewhat agree Neutral

21.- Does the user manual explain the procedure that must be carried out for the notifications review in the application?

Strongly agree Moderately Agree Somewhat agree Neutral

22.- Do you consider that the application should have a guide option to resolve doubts and/or problems regarding the notification visualization?

Strongly agree Moderately Agree Somewhat agree Neutral

23.- Does the application correctly reflect the notifications purpose?

Strongly agree Moderately Agree Somewhat agree Neutral

24.- Is the purpose of the type of notifications in the application clear?

Strongly agree Moderately Agree Somewhat agree Neutral

25.- In the application's notifications option, does it offer help of how to navigate in its elements?

Strongly agree Moderately Agree Somewhat agree Neutral

26.- Is the notification option allowed to return among screens by means of a button and/or using the device's return-button?

Strongly agree Moderately Agree Somewhat agree Neutral

27.- Did the application notify you when a new notification was received?

Strongly agree Moderately Agree Somewhat agree Neutral

28.- Is the notifications status (new, read) reported within the notifications option?

Strongly agree Moderately Agree Somewhat agree Neutral

29.- Do you consider that the notifications in the application are the result of the proper monitoring of the doctor/nurse on the patient's CAPD treatment?

Strongly agree   Moderately Agree   Somewhat agree   Neutral

30.- Do you consider that the application's notifications option has improved the monitoring of your DPCA treatment?

Strongly agree   Moderately Agree   Somewhat agree   Neutral

31.- Has the use of the application's notification option aided communication with the doctor/nurse about the CAPD treatment?

Strongly agree   Moderately Agree   Somewhat agree   Neutral

32.-Does the notification option management always lead to the expected pages without unwanted results (unexpected pages)?

Strongly agree   Moderately Agree   Somewhat agree   Neutral

33.-Does the DPCA exchange record option management always lead to the expected pages without unwanted results (unexpected pages)?

Strongly agree   Moderately Agree   Somewhat agree   Neutral

34.- Would you recommend the application's notifications option to other patients?

Strongly agree   Moderately Agree   Somewhat agree   Neutral

35.- Do you consider it more pleasant and easy to use the notifications option to keep in touch with the doctors/nurses than through other communication means?

Strongly agree   Moderately Agree   Somewhat agree   Neutral

36.- Do you consider that the application's notifications option complements the monitoring of your treatment by the medical and nursing staff?

Strongly agree   Moderately Agree   Somewhat agree   Neutral

37.- Could you provide us with any recommendation to improve the Notifications option of the mobile application?
