# Supplementary material for: Telemonitoring system for patients with chronic kidney disease undergoing peritoneal dialysis: Usability assessment based on a case study
Source: PLoS One. 2018 Nov 6;13(11):e0206600. doi: 10.1371/journal.pone.0206600 (PMC6219778; doi:10.1371/journal.pone.0206600)
Supplement: S4 File — (PDF) [file pone.0206600.s004.pdf]

This questionnaire has the objective of knowing your perspective about the system, as well as the satisfaction of use. The answers you provide will help us to improve the remote monitoring of the treatment of patients on peritoneal dialysis through the system.

Questionnaire: ____ Date: __________________

System user: _____________________________

I: Dialysis Registration Questionnaire

1. In the dialysis record option, when you access the detailed summary or patient's diary, the system informs you if it is doing an operation, e.g., a loading data message?

Strongly agree Moderately Agree Somewhat agree Neutral

2. Does all the information presented in the dialysis record option correspond to the data necessary for monitoring the treatment of your patient?

Strongly agree Moderately Agree Somewhat agree Neutral

3. Can you browse freely in the dialysis record option in the system?

Strongly agree Moderately Agree Somewhat agree Neutral

4. Do dates elements, detailed summary, daily summary, etc. in the dialysis record option, always appear in the same place?

Strongly agree Moderately Agree Somewhat agree Neutral

5. Does the representation of the detailed or daily summary information, displayed in the dialysis record option, allow you to properly analyze the overall balance and/or ultrafiltration?

Strongly agree Moderately Agree Somewhat agree Neutral

6. In the system's dialysis record option, is the type of information that you must enter on the query dates properly indicated?

Strongly agree Moderately Agree Somewhat agree Neutral

7. Does the system guide you properly to view the information required in the dialysis record option?

Strongly agree Moderately Agree Somewhat agree Neutral

8. Is it easy to distinguish the purpose dialysis record option of the system?

Strongly agree Moderately Agree Somewhat agree Neutral

9. Is clear the function performed by all the buttons in the dialysis record option?

Strongly agree Moderately Agree Somewhat agree Neutral

10. Does carrying out the information query in the dialysis record option take 2 to 5 minutes?

Strongly agree Moderately Agree Somewhat agree Neutral

11. In the dialysis record option, is the form for the presentation of patient information visually pleasing?

Strongly agree Moderately Agree Somewhat agree Neutral

12. Is the background color in the dialysis record option adequate?

Strongly agree Moderately Agree Somewhat agree Neutral

13. Is text-font legible in the dialysis record option?

Strongly agree Moderately Agree Somewhat agree Neutral

14. In the dialysis record option, the images and the background colors provide enough contrast with the text?

Strongly agree Moderately Agree Somewhat agree Neutral

15. In the dialysis record option, is it adequately informed if there is an error in the information visualization?

Strongly agree Moderately Agree Somewhat agree Neutral

16. Does the dialysis record option management always lead to the expected pages without unwanted results (unexpected pages)?

Strongly agree Moderately Agree Somewhat agree Neutral

17. In the dialysis record option, are the notification, confirmation and error messages clear?

Strongly agree Moderately Agree Somewhat agree Neutral

18. Does the user manual explain the procedure that must be carried out for the patient's information visualization in the dialysis record option?

Strongly agree Moderately Agree Somewhat agree Neutral

19. Do you consider that the system should have a guide option to solve doubts and/or problems?

Strongly agree Moderately Agree Somewhat agree Neutral

20. Does the information presented in the dialysis record option correctly reflect the monitoring of the patient's treatment?

Strongly agree Moderately Agree Somewhat agree Neutral

21. Is the information presented in the dialysis record option clear?

Strongly agree Moderately Agree Somewhat agree Neutral

22. In the dialysis record option, does it offer help on how to navigate through its elements?

Strongly agree Moderately Agree Somewhat agree Neutral

23. In the dialysis record option allows you to return between screens with a button?

Strongly agree Moderately Agree Somewhat agree Neutral

24. In the dialysis record option, the information presented corresponds to the time periods requested?

Strongly agree Moderately Agree Somewhat agree Neutral

25. Are the steps to carry out the consultations in the dialysis record option clear?

Strongly agree Moderately Agree Somewhat agree Neutral

26. Do you consider that the information presented in the dialysis record option is sufficient for the patient's treatment monitoring?

Strongly agree Moderately Agree Somewhat agree Neutral

27. Do you consider that the data presented in the dialysis record option are adequate to inform doctors/nurses of the treatment of their patient?

Strongly agree Moderately Agree Somewhat agree Neutral

28. Do you consider that the dialysis record option has improved the monitoring of the treatment of your patients?

Strongly agree Moderately Agree Somewhat agree Neutral

29. Has the use of the dialysis record option made it easier for you to access your patient's treatment information?

Strongly agree Moderately Agree Somewhat agree Neutral

30. Does the doctor-patient interaction resulting from the dialysis record option motivate you to continue using the system?

Strongly agree Moderately Agree Somewhat agree Neutral

31. Would you recommend the dialysis record option to other doctors to monitor their APD/CAPD patients?

Strongly agree   Moderately Agree   Somewhat agree   Neutral

32. Do you consider more pleasant and easy to view information about your patient's treatment compared to the traditional method (paper record on behalf of the patient)?

Strongly agree   Moderately Agree   Somewhat agree   Neutral

33. Did you find useful to interact with the dialysis record option for remote monitoring of your patient?

Strongly agree   Moderately Agree   Somewhat agree   Neutral

34. Do you consider that the dialysis record option is a supplement in the monitoring of the treatment of your patients in APD/CAPD?

Strongly agree   Moderately Agree   Somewhat agree   Neutral

35. Do you consider that the dialysis record option has contributed to a more effective/timely patient monitoring?

Strongly agree   Moderately Agree   Somewhat agree   Neutral

36. Could you provide us with any recommendation to improve the dialysis record option of the system?

II: Alerts or notifications questionnaire

1. When visualize alerts generated by patients in the alerts visualization option, the system informs you if it is doing any operation?

Strongly agree Moderately Agree Somewhat agree Neutral

2. In the alerts visualization option, the information presented is consistent with the patient's treatment monitoring?

Strongly agree Moderately Agree Somewhat agree Neutral

3. Does all the information presented in the alerts visualization option correspond to the requested one?

Strongly agree Moderately Agree Somewhat agree Neutral

4. Can you browse freely in the alerts visualization option?

Strongly agree Moderately Agree Somewhat agree Neutral

5. Do dates elements, history, graphs, etc. from alerts visualization always appear in the same place?

Strongly agree Moderately Agree Somewhat agree Neutral

6. Do the structure presented in the alert report and the graph allows you to analyze the behavior of the patient's treatment?

Strongly agree Moderately Agree Somewhat agree Neutral

7. In the alerts visualization option, the dates format required to perform the query is properly indicated?

Strongly agree Moderately Agree Somewhat agree Neutral

8. Is it easy to distinguish the purpose of the alerts visualization option?

Strongly agree Moderately Agree Somewhat agree Neutral

9. Does the function played by all the buttons in the alerts visualization option is clear?

Strongly agree Moderately Agree Somewhat agree Neutral

10. Does carrying out the alert visualization take between 2 and 5 minutes?

Strongly agree Moderately Agree Somewhat agree Neutral

11. In the alerts visualization option, is the representation form of the patient's treatment information visually pleasing?

Strongly agree Moderately Agree Somewhat agree Neutral

12. Is the background color in the or alerts visualization option adequate?

Strongly agree Moderately Agree Somewhat agree Neutral

13. Is the text-font in the alerts visualization option readable?

Strongly agree Moderately Agree Somewhat agree Neutral

14. In the alerts visualization option, do the images and background colors provide enough contrast to the text?

Strongly agree Moderately Agree Somewhat agree Neutral

15. Is the alert visualization option adequately informed if there is an error when requesting the alert report?

Strongly agree Moderately Agree Somewhat agree Neutral

16. Does the alerts visualization option management always lead to the expected pages without unwanted results (unexpected pages)?

Strongly agree Moderately Agree Somewhat agree Neutral

17. Does the user manual explain the procedure that should be carried out for checking alerts of your patient in the alerts visualization option?

Strongly agree Moderately Agree Somewhat agree Neutral

18. Do you think that the system should have a guide option to solve doubts and/or problems about the alerts visualization option?

Strongly agree Moderately Agree Somewhat agree Neutral

19. Is the aim of the system's alert visualization option correctly reflected?

Strongly agree Moderately Agree Somewhat agree Neutral

20. In the system's alerts visualization option, is the presented information clear?

Strongly agree Moderately Agree Somewhat agree Neutral

21. In the system's alerts visualization option, does it offer help on how to navigate through its elements?

Strongly agree Moderately Agree Somewhat agree Neutral

22. In the alerts visualization option, does the system allow you to return between screens with a button?

Strongly agree   Moderately Agree   Somewhat agree   Neutral

23. Are the actions to make a query in the alerts visualization option clear?

Strongly agree   Moderately Agree   Somewhat agree   Neutral

24. Do received alerts (SMS or mail) give you enough information about the treatment (fluid and ultrafiltration characteristics) or its ranges of medical data?

Strongly agree   Moderately Agree   Somewhat agree   Neutral

25. Do you consider that the data presented in the system's visualization option are adequate to inform the doctor/nurse of the patient's treatment?

Strongly agree   Moderately Agree   Somewhat agree   Neutral

26. Do you think that the system's alert visualization option has improved the monitoring of the treatment of your patients?

Strongly agree   Moderately Agree   Somewhat agree   Neutral

27. Has the use of the system's visualization option made it easier for you to control the data generated by the patient's treatment?

Strongly agree   Moderately Agree   Somewhat agree   Neutral

28. Does the doctor-patient interaction resulting from the system's visualization alert option motivate you to carry out accurate communication with your patients?

Strongly agree   Moderately Agree   Somewhat agree   Neutral

29. Would you recommend the system visualization option to other doctors for controlling their patients in APD/CAPD?

Strongly agree   Moderately Agree   Somewhat agree   Neutral

30. Do you consider more efficient to use the system's visualization option to wait for patients to report any condition of their treatment?

Strongly agree   Moderately Agree   Somewhat agree   Neutral

31. Has the system's visualization option been useful for monitoring your patient?

Strongly agree   Moderately Agree   Somewhat agree   Neutral

32. Has the system's visualization option been useful to inform the patient of the action to be taken?

Strongly agree Moderately Agree Somewhat agree Neutral

33. Do you think that the system's visualization option is a complement to the monitoring of patients' treatment?

Strongly agree Moderately Agree Somewhat agree Neutral

34. Could you provide us with any recommendations to improve the system's visualization option?

III: Notifications Questionnaire

1. In the generate notifications options, does the system inform you about the notification status (not synchronized, delivered, etc.)?

Strongly agree Moderately Agree Somewhat agree Neutral

2. Does the notifications generation (recommendations, reminders, appointments, etc.) allow the doctor to provide feedback to the patient about their treatment?

Strongly agree Moderately Agree Somewhat agree Neutral

3. Do you consider that the information added in the notifications is useful to keep the patient informed about their treatment?

Strongly agree Moderately Agree Somewhat agree Neutral

4. Do you consider adequate the generate notifications procedure to inform the patient about their treatment?

Strongly agree Moderately Agree Somewhat agree Neutral

5. In case the answers to the previous questions (3,4) were different from "Strongly agree" please describe the procedure or information that would be desirable to consider:

6. Can you browse freely in the system generate notifications option?

Strongly agree Moderately Agree Somewhat agree Neutral

7. Do the generated notifications for the patient's treatment correspond to those that appear in the general screen of the generate notifications option?

Strongly agree Moderately Agree Somewhat agree Neutral

8. Is the information entered in the generate notifications option adequate to guide the patient about their treatment?

Strongly agree Moderately Agree Somewhat agree Neutral

9. In the generate notifications option, are you promptly informed about the type of information you must enter in the fields (the type of notification, description and expiration date, etc.)?

Strongly agree Moderately Agree Somewhat agree Neutral

10. Is it easy to distinguish the purpose of the system generate notifications option?

Strongly agree Moderately Agree Somewhat agree Neutral

11. Does the notification report allow you to distinguish the different notifications that have been created to the patient?

Strongly agree Moderately Agree Somewhat agree Neutral

12. Does the system allow you to record notification intuitively?

Strongly agree Moderately Agree Somewhat agree Neutral

13. Is the procedure for creating any type of notification similar in the different types of notification?

Strongly agree Moderately Agree Somewhat agree Neutral

14. Does carrying out a notification for a patient in the system take 2 to 5 minutes?

Strongly agree Moderately Agree Somewhat agree Neutral

15. In the generate notifications option, does the system allow you to cancel or perform the required changes?

Strongly agree Moderately Agree Somewhat agree Neutral

16. In the generate notifications option, is the form chosen for the capture and presentation of the information visually pleasing?

Strongly agree Moderately Agree Somewhat agree Neutral

17. Is the background color in the generate notifications option appropriate?

Strongly agree Moderately Agree Somewhat agree Neutral

18. Is the text-font in the generate notifications option readable?

Strongly agree Moderately Agree Somewhat agree Neutral

19. In the generate notifications option, do the images and background colors provide enough contrast with the text?

Strongly agree Moderately Agree Somewhat agree Neutral

20. In the no Internet connection (Wi-Fi) case, does the system inform you about such status?

Strongly agree Moderately Agree Somewhat agree Neutral

21. Does the generate notifications option management always lead to the expected pages without unwanted results (unexpected pages)?

Strongly agree Moderately Agree Somewhat agree Neutral

22. Does the user manual explain the procedure that must be carried out for the visualization and/or record notifications?

Strongly agree Moderately Agree Somewhat agree Neutral

23. Does the information requested in the notifications section correctly reflect its purpose?

Strongly agree Moderately Agree Somewhat agree Neutral

24. In the system generate notifications option, is it offered help about how to navigate in its elements?

Strongly agree Moderately Agree Somewhat agree Neutral

25. In the system generate notifications option, is it possible to return or move between screens with a button?

Strongly agree Moderately Agree Somewhat agree Neutral

26. Are the generated notifications reflected in an appropriate and suitable manner in the notification table?

Strongly agree Moderately Agree Somewhat agree Neutral

27. In the generate notifications option, the notifications' status is distinguished?

Strongly agree Moderately Agree Somewhat agree Neutral

28. Do you consider that the system generate notifications options has improved communication with your patients?

Strongly agree Moderately Agree Somewhat agree Neutral

29. Has the use of the generate notifications options facilitated the monitoring and control of patients?

Strongly agree Moderately Agree Somewhat agree Neutral

30. Would you recommend the generate notifications option to other doctors for monitoring and controlling patients?

Strongly agree Moderately Agree Somewhat agree Neutral

31. Has the generate-notifications-option been useful for the communication with your patients?

Strongly agree Moderately Agree Somewhat agree Neutral

32. Do you consider the generate notifications option is an augmentation for the monitoring and control of the patients in APD/CAPD treatment?

Strongly agree   Moderately Agree   Somewhat agree   Neutral

33. Could you provide us with any recommendation to improve the system generate notifications option?

34. Regarding the different system's options, which one do you consider more important and could you propose overall recommendations to improve the system?
